# An Inverse Optimal Control Approach to Explain Human Arm Reaching Control Based on Multiple Internal Models

**DOI:** 10.1038/s41598-018-23792-7

**Published:** 2018-04-03

**Authors:** Ozgur S. Oguz, Zhehua Zhou, Stefan Glasauer, Dirk Wollherr

**Affiliations:** 1 Chair of Automatic Control Engineering (LSR), Department of Electrical and Computer Engineering, Technical University of Munich, Munich, 80333 Germany; 2 Department of Neurology, Ludwig-Maximilian-University Munich, Munich, 81377 Germany

## Abstract

Human motor control is highly efficient in generating accurate and appropriate motor behavior for a multitude of tasks. This paper examines how kinematic and dynamic properties of the musculoskeletal system are controlled to achieve such efficiency. Even though recent studies have shown that the human motor control relies on multiple models, how the central nervous system (CNS) controls this combination is not fully addressed. In this study, we utilize an Inverse Optimal Control (IOC) framework in order to find the combination of those internal models and how this combination changes for different reaching tasks. We conducted an experiment where participants executed a comprehensive set of free-space reaching motions. The results show that there is a trade-off between kinematics and dynamics based controllers depending on the reaching task. In addition, this trade-off depends on the initial and final arm configurations, which in turn affect the musculoskeletal load to be controlled. Given this insight, we further provide a discomfort metric to demonstrate its influence on the contribution of different inverse internal models. This formulation together with our analysis not only support the multiple internal models (MIMs) hypothesis but also suggest a hierarchical framework for the control of human reaching motions by the CNS.

## Introduction

Human motor control shows a remarkable ability to regulate stereotypical human motions that are observed for a broad range of tasks in daily life. On the one hand, an influential idea discussed in literature is that, the central nervous system (CNS) utilizes internal models, which hypothetically represent the control of dynamics and kinematics of movement, to achieve such an efficient motor behavior^1,2^. On the other hand, another major line of work hypothesizes that, after an initial learning phase, the stereotypical human motions are approximately optimal with respect to an unknown criterion. Hence, modeling of human motor behavior has been posed as an optimal control problem and several cost functions have been proposed^3–7^. If one interprets an optimal control problem with different cost functions as different control models, the combination of them corresponds to the multiple inverse internal models formulation. Depending on the task, the combinations of those models can be adapted accordingly. If we assume the space of 3D free space motion control models is represented as a combination of internals models within the cerebellum, then the follow-up question is whether there exists a hierarchical control where the contribution of those different internal models are regulated. Analysis of such a hierarchical control mechanism can improve our understanding and interpretation of how the CNS might be internally representing and utilizing some critical metrics to implicitly control stereotypical movements for a multitude of tasks. In this paper, we search for such internal models and their combinations to support the MIM hypothesis by focusing on the arm reaching motions in a wide range of the 3D space. Subsequently, how those internal models are combined for arm motions and why their contributions change depending on the reaching task are investigated.

Searching for the physiological foundations of motor control and internal models, various experiments are discussed in the literature^8–10^. Resulting hypotheses are that the posterior parietal cortex is updating the motor plan, while the cerebellum might capture the feedforward control signals. Further structures of the human nervous system might contain the state estimator and comparator^9^. In this regard, feedforward and feedback control mechanisms along with learning and adaptation processes for motor control has been studied extensively^11–16^. These results suggest that computational approaches to motor learning and control should include two separate performance errors rather than one^17^, and that the brain learns multiple internal models that can be combined as required by the circumstance^18^. Several studies investigated whether multiple internal models could be learned concurrently, and switched depending on the task^19,20^. It was shown that two learned models could be additively combined^21^, and a modular controller selection architecture was proposed which relied on a linear combination of the outputs of the multiple inverse internal models^22^. However, verification of those composite models for a broad range of tasks is missing and how they are combined and controlled efficiently by the CNS are still unclear.

Before even identifying multiple models utilized by the CNS, finding a single internal model is a challenging computational problem. A common feature of motor control is that the task requirements can be met by infinitely many diverse movements. Thus, providing only the boundary conditions of the motion for given dynamics leads to an ill-defined problem. The ambiguity caused by this problem can be resolved if an optimality principle is applied. Accordingly, the basis of many scientific theories on human motor control is formed by optimality principles. A large number of models of open-loop motor control exist and each model claims to describe human motion, but several models are incompatible with others. The starting point for the derivation of a cost function are characteristics of the human arm movements and the human as a biological entity. Human motor control has been speculated to minimize the sequence of control signals^23–25^, or limb states^26^. These minimization strategies are related to physiological and task variables such as smoothness of the hand path^3,4^, accuracy^5,27^ or error and effort^28–32^. However, these single models appear to be not descriptive enough for a broader range of tasks. Hence, recent studies focus on finding a combination of such models. These methods solve an Inverse Optimal Control (IOC) problem where the contribution of different optimal control models are computed^33–35^. The best combination of models that results in a trajectory as similar as to the recorded human motion data is identified through iteratively comparing the calculated motion trajectory with the observation, while still satisfying all individual control models w.r.t. the dynamical system and the constraints. IOC formulation describes human motion better than the previous single models, yet it forms a more complex computational problem, which emphasizes the importance of a better understanding of why and how those models are efficiently utilized by the CNS.

**Figure 1:**
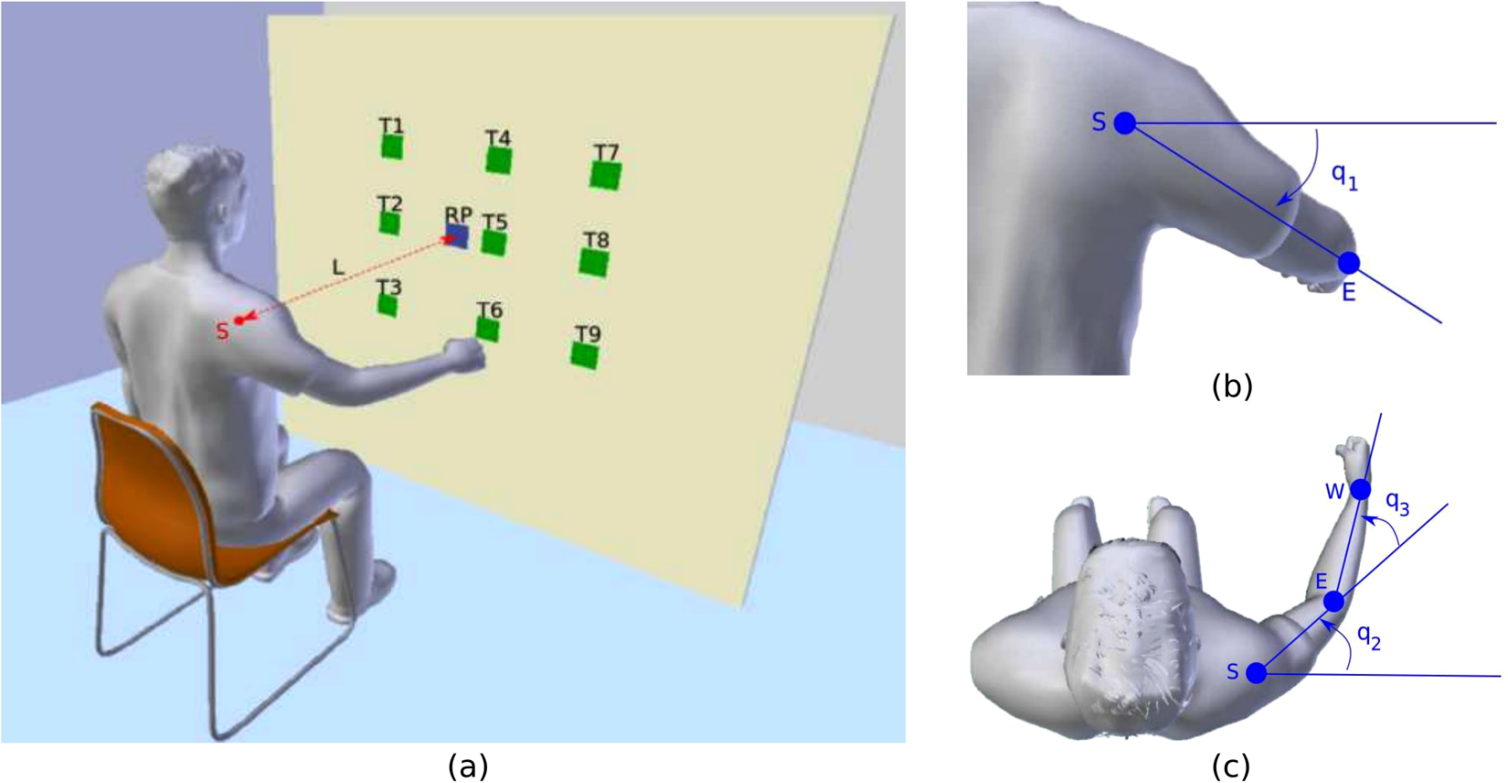
Experimental setup. (**a**) Experimental overview. T1 to T9 indicate nine target areas. RP stands for the reference point, which is used to adjust subject’s position. The distance L between the center of the shoulder joint S and the RP is selected as 80% of the total arm length. Subjects are required to reach nine target areas from nine different starting arm postures. (**b**–**c**) Three rotations defined in our arm model. S, E, W are the positions of shoulder, elbow, and wrist joints, respectively. When the arm is in full stretched out posture, *q*_1_, *q*_2_ and *q*_3_ all have zero rotations.

In this study, we focus on finding evidence for MIMs hypothesis and understanding how these models are efficiently combined by the CNS. Considering that internal models mimic the transformations between system states, motor commands, and sensory signals, an optimal control problem (OCP) can be regarded as an inverse internal model by providing the necessary control signals to carry out the reaching task^2,17,36^. Our hypothesis is that, if a comprehensive analysis is done on a rich set of human motion, the change in the contribution of the underlying motor control structures can be observed in a way to reveal the factors that explain such an adjustment. We limit ourselves to examining simple, 3D free-space arm reaching movements. Simple movements can be considered to make up a basis set of which more complicated movements are composed^37–39^, and as such, studying them provides insight into the control of more complex movements. We devised an experiment in which a wide range of 3D reaching motions are recorded (Fig. 1). For the arm reaching task we focused, a single forward model, i.e. the arm dynamics, is paired with multiple inverse internal models, i.e. the composite of OCP where each OCP is associated with a specific cost function.

This paper presents both a comprehensive optimality analysis on human arm reaching motions in 3D space and a trade-off between dynamics and kinematics based controllers depending on the reaching motion type. The main contributions of our work are three-fold:

- Multiple inverse internal models, represented as a combination of different controllers, are discovered to be collectively controlling the 3D free-space arm reaching movements.
- We explain quantitatively how the contribution of these inverse internal models change w.r.t. the task parameters.
- Finally, a hierarchical control model that regulates MIMs for controlling reaching motions is proposed.

## Results

We have three main results, which support our hypothesis on hierarchical control of multiple internal models within the CNS. First, there are discernible behavioral differences, such as velocity profiles and reaching time, depending on the reaching motion type. Such differences in movement features signal the existence of different motion control structures. Second, we identified that the combination of different optimal controllers explains a broad range of human reaching motions in 3D free space better than a single one. As each OCP with a given cost function defines a single control mechanism and thus a single inverse internal model, having several controllers supports the multiple inverse internal models component of the multiple internal models (MIMs) hypothesis. Finally, the combination of those internal models changes depending on the initial and final arm postures. This dependency, we claim, is resolved by the CNS by a hierarchical control mechanism to actively coordinate the contribution of those independent internal models. To this end, we propose a metric, that is a function of motion features, as a higher level control parameter since it can describe the dependence of model contributions to the motion parameters.

### Reaching motion behavior analysis

#### Initial and final postures

**Figure 2:**
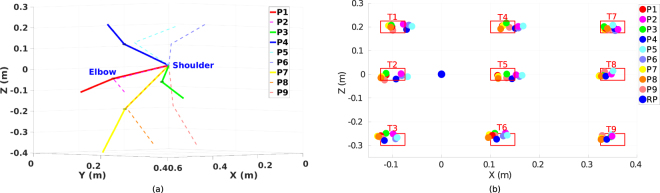
Example of the experimental design. (**a**) An illustration of nine initial arm postures. P1 to P3 are three postures in the middle level, while P4 to P6 and P7 to P9 are postures in the upper and lower level, respectively. Differences between P1 and P2, P4 and P5, P7 and P8 are that *q*_3_ increases while *q*_2_ remains constant, while between P1 and P3, P4 and P6, P7 and P9, *q*_3_ remains constant and *q*_2_ increases. (**b**) Positions of the nine target areas and the reference point (RP) on the board surface. Target areas are squares with side length equals to 5 cm. An example of the reach endpoints for subject 3 is also presented. P1 to P9 indicate the initial arm posture for each corresponding reach endpoint.

The experimental task is demonstrated in Fig. 1. Every subject starts the reaching tasks from nine different initial arm postures and reaches nine target areas (9 initial postures × 9 targets = 81 reaching tasks). The initial postures are selected as different combinations of three joint angles, where *q*_1_ has three values corresponding to the upper, middle, down arm postures, *q*_2_ and *q*_3_ have two values which stand for the zero rotation and a rotation approximately has 40°, respectively. The actual initial joint angles averaged from all subjects’ data are listed in Table 1. The results show that for the same reaching task, participants share similar initial joint angle configurations which satisfies our experimental design. An illustration of the initial arm postures and the corresponding reach endpoints on the board surface for a representative subject (subject 3) is presented in Fig. 2.

**Table 1:** Actual initial and final joint angle configurations calculated among all 15 subject’s data.

Posture	*q*_1*s*_(°)	*q*_2*s*_(°)	*q*_3*s*_(°)	Target	*q*_1*e*_(°)	*q*_2*e*_(°)	*q*3*e*(°)
P1	11.0 ± 5.0	6.6 ± 4.7	12.7 ± 3.5	T1	−10.3 ± 3.6	82.0 ± 6.9	23.7 ± 7.2
P2	11.2 ± 5.7	8.8 ± 9.5	33.4 ± 6.5	T2	7.2 ± 4.2	80.5 ± 7.6	29.4 ± 10.2
P3	11.9 ± 3.7	31.9 ± 5.8	13.2 ± 3.7	T3	27.5 ± 4.7	83.5 ± 7.0	24.5 ± 9.1
P4	−22.3 ± 5.2	12.5 ± 5.2	14.1 ± 3.9	T4	−10.2 ± 3.7	66.6 ± 6.1	19.0 ± 5.7
P5	−23.5 ± 5.7	15.8 ± 6.4	37.9 ± 7.5	T5	6.7 ± 3.8	64.3 ± 7.0	24.0 ± 8.7
P6	−22.9 ± 5.3	37.3 ± 7.9	16.1 ± 5.1	T6	26.6 ± 4.3	66.5 ± 7.0	20.4 ± 8.3
P7	42.2 ± 6.2	7.0 ± 7.4	12.3 ± 4.6	T7	−9.1 ± 3.4	55.0 ± 5.0	14.4 ± 4.5
P8	40.2 ± 4.4	7.1 ± 5.3	35.4 ± 5.6	T8	5.2 ± 3.4	53.9 ± 5.4	14.9 ± 5.4
P9	35.4 ± 5.1	36.1 ± 5.6	10.1 ± 5.8	T9	23.6 ± 3.6	54.5 ± 5.9	13.0 ± 5.4

Mean values and the corresponding standard derivations are presented. P1 to P9 are nine different initial arm postures. T1 to T9 indicate nine target areas. (*q*_1*s*_, *q*_2*s*_, *q*_3*s*_) are the three initial joint angles while (*q*_1*e*_, *q*_2*e*_, *q*_3*e*_) are the final joint angles for each target area.

Since our focus is on stereotypical arm reaching characteristics that are observed on the trajectory level, and the corresponding feedforward controllers that might result in such trajectories, the experiment conducted is designed to allow the subjects to reach their hands to a region rather than a single point target. To investigate whether the initial arm posture has influence on the selection of reach endpoints and final arm postures, one-way repeated measures ANOVAs (using SPSS statistics) are conducted separately for each target area. Corresponding *p*-values are presented in Table 2. The results show that, no significant difference of the initial arm posture conditions on the Cartesian coordinates of the reach endpoints *x*, *y*, *z* is found (*p*_*x*, *y*, *z*_ > 0.05 for all target areas). This suggests that, the selection of reach endpoints, which exhibit elliptical distributions inside each target area, is unaffected by the initial arm postures.

**Table 2:** One-way repeated measures ANOVAs of the initial arm posture conditions on the Cartesian coordinates of the reach endpoints *x*, *y*, *z* and final arm joint angle configurations (*q*_1*e*_, *q*_2*e*_, *q*_3*e*_) separately for each target area. Due to the space limitation, only *p*-values are given here.

Target	*p*-values					
	*x*	*y*	*z*	*q*_1*e*_	*q*_2*e*_	*q*3*e*
T1	0.212	0.803	0.999	0.902	0.041	0.003
T2	0.280	0.793	0.999	0.938	0.041	0.066
T3	0.324	0.717	0.999	0.731	0.053	0.057
T4	0.422	0.761	0.999	0.654	0.004	0.001
T5	0.619	0.556	0.999	0.946	0.004	0.043
T6	0.609	0.447	0.999	0.825	0.017	0.071
T7	0.591	0.738	0.999	0.105	0.007	0.007
T8	0.624	0.496	0.999	0.474	0.004	0.003
T9	0.634	0.349	0.999	0.394	0.009	0.013

However, due to the redundancy of arm kinematics, given only the target area might result in numerous possible final arm joint angle configurations which can satisfy the task requirements. The results of one-way repeated measures ANOVAs on the three final joint angles also support this possibility. The redundancy mainly happens in the ulnar/radial rotation of the shoulder joint *q*_2*e*_ and the extension/flexion rotation of the elbow joint *q*_3*e*_, which are found to be influenced by the initial arm postures (${p}_{{q}_{2e}} < 0.05$ except target three, *p*_3*e*_ < 0.05 except target two, three and six), whereas the elevation/depression rotation of the shoulder joint *q*_1*e*_ remains irrelevant to the initial arm postures (*p*_1*e*_ > 0.05 for all target areas).

These results may also be due to the fact that, in our experimental design, participants are asked to avoid using the internal/external rotation of the shoulder joint during the reaching movements, and thus the height of reach endpoints can only be determined by *q*_1_. By specifying the height of target areas, the choice of *q*_1*e*_ is quite limited, so that no significant influence of the initial arm posture on *q*_1*e*_ can be found. In contrast, same target area can be reached by different combinations of *q*_2_ and *q*_3_ which exhibit similar hand positions in *x*,*y* direction. As a consequence, the initial joint angle configurations affect the selection of final arm postures, as subjects show different tendencies in this selection. Some of them prefer a straight arm posture with low rotation in the elbow joint, while others would like to keep the rotation of the elbow joint the same as the initial configuration. The averaged final arm postures among all subjects’ data are also presented in Table 1.

#### Movement duration

**Figure 3:**
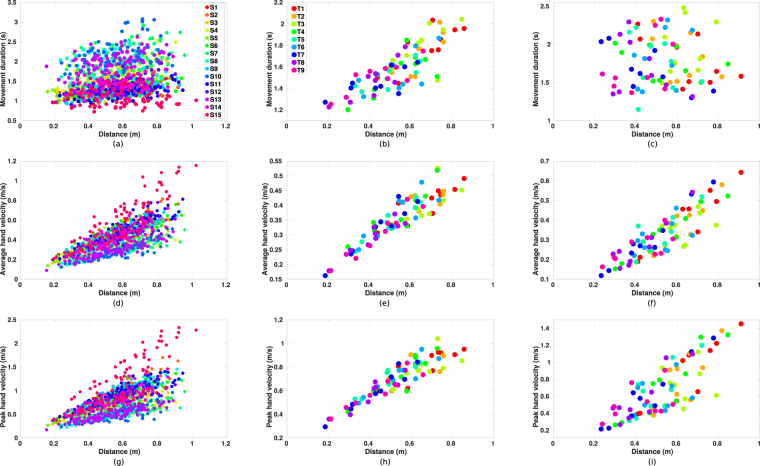
Relationship between target distance and movement duration, average hand velocity, peak hand velocity. (**a**) The distribution of target distance and movement duration for all subjects’ data. Points are grouped by 15 subjects as from S1 to S15. (**b**) The relationship between target distance and movement duration for subject 3. Points are grouped by the target areas for the corresponding reaching tasks. (**c**) The relationship between target distance and movement duration for subject 8. (**d**–**f**) The distribution of target distance and average hand velocity for all subjects’ data, subject 3, and subject 8, respectively. (**g**–**i**) The distribution of target distance and peak hand velocity for all subjects’ data, subject 3, and subject 8, respectively.

The movement durations averaged among all subjects’ data of reaching tasks starting from initial arm posture one are presented in Table 3. The full table of all 81 tasks is given in the supplementary material. We first investigate the relationship between the movement duration and the target distance which is defined as the distance between the initial hand position and the reach endpoint. The distribution of target distances and movement durations of all subjects’ data is presented in Fig. 3a. The result of correlation analysis (using MATLAB *corrcoef* function) indicates that there is a weak correlation between target distance and movement duration (*r* = 0.245, *p* < 0.001) among all the data. However, different behaviors are observed among subjects. Two representative subjects, one with strong correlation (subject 3, in Fig. 3b, *r* = 0.815, *p* < 0.001) and another without correlation (subject 8, in Fig. 3c, *r* = −0.069, *p* = 0.543), are presented. This indicates that, different subjects may have different motor control strategies in their natural reaching movements, i.e. some prefer a task-dependent movement duration while others adapt the velocity so that the durations of different reaching tasks are on the same level. Individual analysis shows that most of the subjects adapt their velocities with respect to the target distance (see supplementary material), hence in general the observed correlation is weak.

**Table 3:** Movement duration and kinematic features of reaching tasks starting from initial arm posture one towards nine target areas.

Target	Movement duration (s)	Target distance (m)	Average hand velocity (m/s)	Peak hand velocity (m/s)
T1	1.73 ± 0.36	0.813 ± 0.044	0.53 ± 0.11	1.00 ± 0.23
T2	1.63 ± 0.35	0.775 ± 0.051	0.54 ± 0.12	1.04 ± 0.30
T3	1.70 ± 0.37	0.779 ± 0.058	0.52 ± 0.12	0.98 ± 0.26
T4	1.57 ± 0.32	0.689 ± 0.043	0.48 ± 0.10	0.91 ± 0.22
T5	1.49 ± 0.30	0.645 ± 0.050	0.48 ± 0.11	0.88 ± 0.22
T6	1.57 ± 0.37	0.650 ± 0.060	0.47 ± 0.12	0.86 ± 0.22
T7	1.52 ± 0.30	0.574 ± 0.037	0.41 ± 0.09	0.76 ± 0.18
T8	1.44 ± 0.25	0.535 ± 0.045	0.40 ± 0.08	0.72 ± 0.18
T9	1.47 ± 0.25	0.533 ± 0.052	0.39 ± 0.06	0.72 ± 0.19

Mean values and standard derivations are presented. The data is averaged among all subject’s data.

In addition, two-way repeated measures ANOVAs are performed to investigate whether the initial arm posture and target area conditions have influence on the movement duration. The results show significant differences both in target area conditions (*F*(8, 112) = 18.5, *p* < 0.001) and the interaction between target area and initial posture conditions (*F*(64, 896) = 4.4, *p* < 0.001). However, the initial posture conditions exhibit no significant difference (*p* = 0.084). This suggests that, the movement duration is partially affected by the target distance, but in the meantime is also influenced by other reaching motion parameters, e.g. arm configurations.

#### Kinematic features

**Figure 4:**
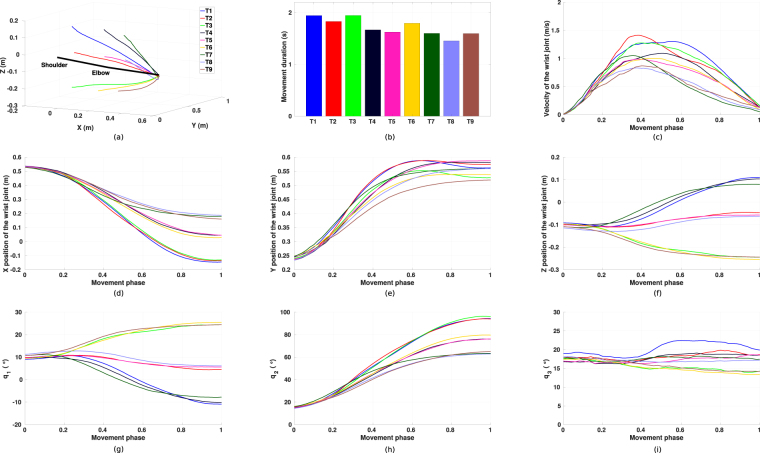
Examples of nine reaching tasks from initial arm posture one for subject 3. The subplots show (**a**) end-effector trajectories, (**b**) movement durations, (**c**) velocity of the wrist joint, where the trajectories are presented with their movement phase, which starts at 0 and ends at 1, (**d**–**f**) *x*, *y*, *z* coordinates of the wrist joint, (**g**–**i**) trajectories of the three joint angles.

All subjects exhibit similar kinematic features during the reaching motions. The hand velocity and angular velocity profiles are bell-shaped and demonstrate classical patterns in point-to-point reaching tasks. A partial table of the kinematic features averaged among all subjects is presented in Table 3 (see supplementary material for full data). Typical kinematic features of a representative subject (subject 3) are presented in Fig. 4. To further investigate the influence of initial arm posture and target area conditions on the kinematic features, two-way repeated measures ANOVAs are conducted on the average hand velocity and the peak hand velocity. Significant differences are found in the initial posture conditions (*F*_*average*_(8, 112) = 14.3, *p*_*average*_ < 0.001; *F*_*peak*_(8, 112) = 9.5, *p*_*peak*_ < 0.001), the target area conditions (*F*_*average*_(8, 112) = 107.3, *p*_*average*_ < 0.001; *F*_*peak*_(8, 112) = 87.1, *p*_*peak*_ < 0.001) and the interaction between initial posture and target area conditions (*F*_*average*_(64, 896) = 31.1, *p*_*average*_ < 0.001; *F*_*peak*_(64, 896) = 37.9, *p*_*peak*_ < 0.001).

Considering the target distance as another parameter of the reaching task, similar results are also found in the correlation analysis between the target distance and the velocities. As explained in the movement duration part, subjects would adapt their reaching velocity according to the target distance, hence relative stronger correlations between the velocities and the target distance are found (*r*_*average*_ = 0.694, *p*_*average*_ < 0.001; *r*_*peak*_ = 0.654, *p*_*peak*_ < 0.001). Corresponding distributions of the same two subjects are presented in Fig. 3. It can be observed that, both subjects demonstrate good correlations between the velocities and the target distance (Fig. 3e, *r*_*average*_ = 0.914, *p*_*average*_ < 0.001, Fig. 3h, *r*_*peak*_ = 0.908, *p*_*peak*_ < 0.001 for subject 3; Fig. 3f, *r*_*average*_ = 0.864, *p*_*average*_ < 0.001, Fig. 3i, *r*_*peak*_ = 0.786, *p*_*peak*_ < 0.001 for subject 8). Note that, unlike most of the subjects, the movement durations of subject 3 do not decrease even though the dependency is reported between the velocity and the distance. This may be due to a different path planning strategy adapted by the subject.

All these adaptations, including the final arm postures, velocity profiles and hand trajectories, suggest that the reaching motion behavior changes depending on the reaching task. Thus, it is possible that the CNS also changes its motor control strategy based on the reaching motion parameters. To further investigate the underlying control strategy of reaching motions, an analysis based on the inverse optimal control results is presented in the following sections.

### Identification of multiple internal models

Internal models are neural mechanisms that control a specific property of human movement by imitating the input/output characteristics of the motor apparatus. However, even simple human motions, such as reaching a cup, require a complex system to control the kinematics and dynamics properties of the human arm. To identify the components of such a control mechanism, we first examine the existence of multiple internal models for a broad range of human reaching movements. In our experiment, stereotypical 3D free-space arm reaching task is the focus, and accordingly, in our formulation there is a single forward internal model, i.e. the arm dynamics model, that solves for the system states given the control input, but it is paired with different inverse models. Inverse optimal control results show that the combinations of optimal controllers, i.e. multiple inverse models, result in a better fit than a single optimal control based solutions. Additionally, the contribution of internal models is found to be dependent on the movement type.

#### Reconstruction errors

By means of forward optimal control, motion trajectories are simulated to calculate the reconstruction errors of the solutions with respect to the single model and the composite model control strategies. In the single model cases, the single basic cost functions (hand jerk, joint angle jerk, torque change and energy, see method section) are used individually to predict human motion trajectories for all the reaching tasks we have considered. Then, in the composite model case, by using the optimal composite cost function obtained from the IOC calculations, we simulate the reaching trajectories. Thereafter, the reconstruction errors of those single models and the composite model are compared by computing the distance error between the simulated trajectories and the observations.

**Figure 5:**
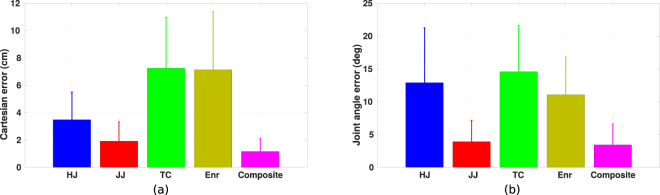
Reconstruction errors normalized with the number of data points. (**a**) The Cartesian errors of reconstructions by using the minimum hand jerk (HJ), minimum joint angle jerk (JJ), minimum torque change (TC), minimum energy (Enr) and the composite model (Composite), respectively. (**b**) The joint angle errors of reconstructions.

Two different kinds of errors are measured through the dynamic time warping, one is the end-effector Cartesian coordinate error, another is the joint angle error. As presented in Fig. 5a, the composite model can simulate the reaching motions with smaller end-effector trajectory error (1.2 ± 0.9 cm) compared to all other single models (hand jerk: 3.5 ± 2.0 cm, joint angle jerk: 1.9 ± 1.4 cm, torque change: 7.3 ± 3.7 cm, energy: 7.1 ± 4.2 cm). One-way repeated measures ANOVAs are performed separately on different single models and the composite model. The results demonstrate significant differences between the composite model and each other single model (hand jerk vs composite: *F*(1, 1200) = 2080.9, *p* < 0.001; joint angle jerk vs composite: *F*(1, 1200) = 863.2, *p* < 0.001; torque change vs composite: *F*(1, 1200) = 3641.0, *p* < 0.001; energy vs composite: *F*(1, 1200) = 2732.8, *p* < 0.001). Similar results can also be observed in the joint angle errors, which are illustrated in Fig. 5b. One-way repeated measures ANOVAs also show significant differences between the composite model and each single model (hand jerk vs composite: *F*(1, 1200) = 1723.4, *p* < 0.001; joint angle jerk vs composite: *F*(1, 1200) = 111.0, *p* < 0.001; torque change vs composite: *F*(1, 1200) = 4170.8, *p* < 0.001; energy vs composite: *F*(1, 1200) = 3175.3, *p* < 0.001). Even the joint angle trajectory is not considered in the presented IOC formulation (see method section), the composite model still exhibits better performance in describing the angular trajectories with an average error of 3.4 ± 3.1° compared to other single models (hand jerk: 12.9 ± 8.4°, joint angle jerk: 3.9 ± 3.2°, torque change: 14.6 ± 7.1°, energy: 11.1 ± 5.8°). The smaller reconstruction errors of the composite model suggest that, rather than a single model, the CNS may use multiple models to control its reaching motions.

For the model fitting analysis, Akaike and Bayesian information criterion (AIC and BIC, respectively) were computed for the models with 2, 3, and 4-cost function combinations. A representative subject’s data is used to compute the composite models for each combination. The composite model with the 2-cost functions used joint-angle-jerk (kinematics) and torque-change (dynamics), whereas for the 3-cost function model energy (dynamics) cost was also included together with the previous two. The resulting AIC and BIC comparisons favored the 4-cost function model compared to the other two models. The end-effector Cartesian coordinate error was also computed to compare these models in terms of their accuracy in representing the human behavior. This analysis demonstrated a statistically significant difference (for both 2- vs. 4-cost function model, and 3- vs. 4-cost function model, *p* < 0.001), favoring the 4-cost function composite model (see supplementary material).

#### Contribution of models

Having identified that the composite model as the controller describes human reaching motion behaviors better than the single models, we also investigate whether the combination of different basic models changes depending on the reaching tasks. Two-way repeated measures ANOVAs are performed separately on the contribution of hand jerk *α*_*HJ*_, joint angle jerk *α*_*JJ*_, torque change *α*_*TC*_ and energy *α*_*Enr*_ controller models with respect to the initial arm posture and target area conditions. The results show significant differences both in the target area conditions for all four base models (*F*_*HJ*_(8, 112) = 6.8, *p*_*HJ*_ < 0.001; *F*_*JJ*_(8, 112) = 3.9, *p*_*JJ*_ < 0.001; *F*_*TC*_(8, 112) = 10.6, *p*_*TC*_ < 0.001; *F*_*Enr*_(8, 112) = 15.0, *p*_*Enr*_ < 0.001), as well as in the interaction between the initial arm posture and target area conditions (*F*_*HJ*_(64, 896) = 2.6, *p*_*HJ*_ < 0.001; *F*_*JJ*_(64, 896) = 1.5, *p*_*JJ*_ < 0.01; *F*_*TC*_(64, 896) = 1.4, *p*_*TC*_ < 0.05; *F*_*Enr*_(64, 896) = 4.4, *p*_*Enr*_ < 0.001). For the initial arm posture conditions, except the contribution of joint angle jerk controller (*p* = 0.199), significant differences are found for other three basic models (*F*_*HJ*_(8, 112) = 17.4, *p*_*HJ*_ < 0.001; *F*_*TC*_(8, 112) = 5.3, *p*_*TC*_ < 0.001; *F*_*Enr*_(8, 112) = 12.6, *p*_*Enr*_ < 0.001). The reason for *α*_*JJ*_ being not affected by the initial posture conditions may be that *α*_*JJ*_ is more consistent in different reaching tasks. Particularly, the joint angle jerk controller plays a dominant role in the motor control of reaching motions, which has an average value of *α*_*JJ*_ = 0.45 ± 0.14, while other three basic controllers have smaller contributions (*α*_*HJ*_ = 0.31 ± 0.18;*α*_*TC*_ = 0.09 ± 0.12;*α*_*Enr*_ = 0.15 ± 0.16). This is supported by the reconstruction errors exhibited in Fig. 5, where the joint angle jerk model can simulate the trajectories with smaller errors compared to other three models, and the results are also similar to the composite model. This can be explained by the fact that, the recorded reaching motions mostly have bell-shaped velocity profiles, while the optimal control solution to the joint angle jerk cost function also produces bell-shaped velocities, hence the joint angle jerk based controller can represent the reaching motions with better performance.

In addition, we also group the four base controllers into two types: the dynamics and the kinematics related control models (see method section). Two-way repeated measures ANOVAs also indicate significant differences in the contribution of dynamics based controllers *α*_*Dyn*_ with respect to the initial arm postures conditions (*F*(8, 112) = 15.1, *p* < 0.001), the target area conditions (*F*(8, 112) = 11.7, *p* < 0.001) and the interaction between the initial arm posture and target area conditions (*F*(64, 896) = 3.03, *p* < 0.001). The contribution of kinematics based controllers *α*_*Kin*_ demonstrates the same results since the sum of *α*_*Dyn*_ and *α*_*Kin*_ equals to one. To simplify the analysis and reduce the dimension of possible variables, we focus on analyzing the contribution of dynamics based controllers in the following section.

### Hierarchical control of internal models

As the contribution of dynamics based controllers is found to be influenced by the initial arm posture and target area conditions, we further investigate whether this influence follows a certain criterion. For each given reaching task, the motion parameters can be defined as the initial arm posture with three initial arm joint angles ***q***_*s*_ = (*q*_1*s*_, *q*_2*s*_, *q*_3*s*_) and the final arm posture (represents target area) with the corresponding final arm joint angles ***q***_*e*_ = (*q*_1*e*_, *q*_2*e*_, *q*_3*e*_). The purpose of this section is to identify if there is a relationship between the contribution of dynamics based controllers and the motion parameters.

#### Principal component analysis

**Figure 6:**
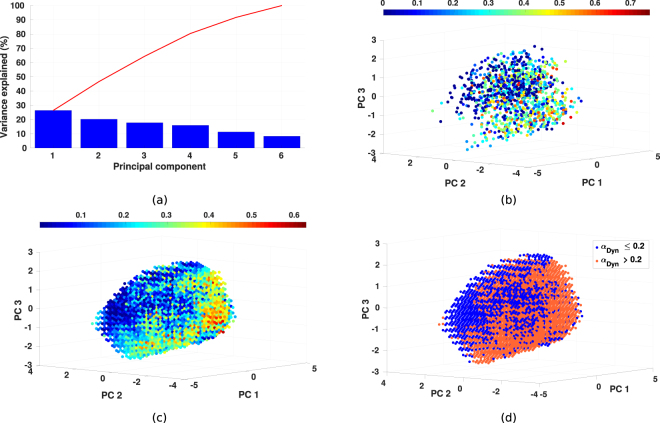
Results of principal component analysis (PCA). (**a**) Variance explained by each principal component. Red line indicates the total explained variances summed up from corresponding principal components. (**b**) Original distribution of *α*_*Dyn*_ with respect to the first three principal components by using all subjects’ data. PC 1 to PC 3 indicate the first three principal components, the color of the points stands for the contribution of dynamics based controllers. (**c**) The distribution of *α*_*Dyn*_ after the interpolation by using all subjects’ data. (**d**) Two groups of the points divided from Fig. 6c by using the threshold as *α*_*Dyn*_ = 0.2.

As each reaching task is defined through six joint angles, due to this high dimensionality, it is not straightforward to determine whether the relationship between the motion parameters and the contribution of dynamics related controllers exists. Hence, we first use the principal component analysis (PCA) to transfer the original motion parameters to the principal components (PCs) by using all subjects’ data. For visualization purposes, we select the first three PCs, which only explain 64% of the variance (Fig. 6a), as a new motion parameter representation for each reaching task, and then investigate the distribution of *α*_*Dyn*_ with respect to these PCs.

The original IOC results of all subjects’ data are illustrated in Fig. 6b, where the *x*, *y*, *z* coordinates of each data point are the corresponding values of the first three PCs, and the color indicates the value of *α*_*Dyn*_. Due to the infeasibility of the global optimum in the IOC formulation^35^, the results of *α*_*Dyn*_ prone to noise. Besides, the insufficient data amount (9 initial postures × 9 targets × 15 subjects = 1215 motions in total) also limits the determination of the relationship between PCs and *α*_*Dyn*_. In order to overcome this issue and have a better visualization, the original data is interpolated with a 3D interpolation method (using MATLAB *griddata* function) and the corresponding results are presented in Fig. 6c. It is observed from the given perspective of the figure that, upper-left points on the point cloud usually have smaller *α*_*Dyn*_, while the points with high values of *α*_*Dyn*_ mostly appear in the opposite region. A more clear illustration is presented in Fig. 6d, where the data points are divided into two groups with the threshold as *α*_*Dyn*_ = 0.2. Hence through visual inspection, even it only represents a subspace of 6 PCs, a trend that indicates a dependence between the motion parameters of the reaching task and the distribution of *α*_*Dyn*_ is observed. In addition, as the sum of *α*_*Kin*_ and *α*_*Dyn*_ is one, the observed distribution suggests a trade-off between the kinematics and dynamics related controllers based on the type of the reaching task.

Note that, the first three PCs account only for 64% of the total variance. Thus, in order to explain the distribution more accurately, we opt for using all six motion parameters to identify a criterion, which we refer to as *discomfort* metric, in order to describe the distribution of *α*_*Dyn*_.

#### Discomfort metric

As found in a recent study, for reaching motions the contribution of interaction torque to net torque changes depending on the load on the arm^40^. This result hints that the distribution of *α*_*Dyn*_ may be related to the musculoskeletal loading of the arm during the movements. Considering the musculoskeletal loading as the criterion to describe the discomfort of the reaching motions^41,42^, the fully stretched down arm posture can be treated as the most comfortable posture. Then the more rotations the arm requires to execute the reaching task from the fully stretched down posture, the more uncomfortable the motion is. Based on this, we propose a discomfort metric (see method section) to explain the distribution of *α*_*Dyn*_.

**Figure 7:**
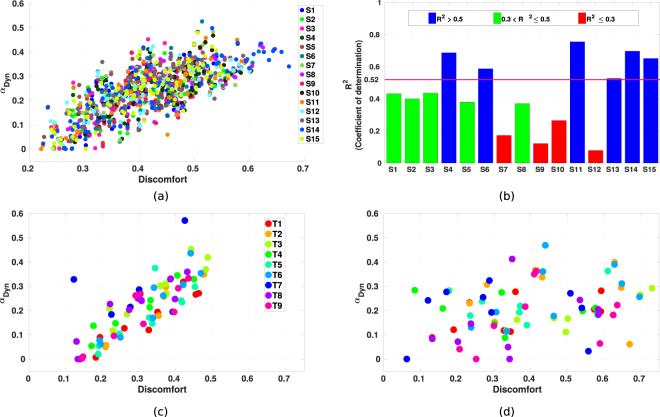
Results of the discomfort metric. (**a**) The distribution of *α*_*Dyn*_ and the discomfort values calculated from all subjects’ data. The points are grouped by different subjects as from S1 to S15. (**b**) The coefficient of determination between *α*_*Dyn*_ and the discomfort values calculated by using each subject’s data separately. The line indicates the overall coefficient of determination from all subjects’ data presented in Fig. 7a (*R*^2^ = 0.52). (**c**,**d**) The distribution of *α*_*Dyn*_ and the discomfort values for subject 15 and subject 12, respectively. The points are grouped by the target areas for the corresponding reaching tasks.

We first investigate the overall behavior by using all subjects’ data. Due to the same noise issue explained in previous part, during the calculation of discomfort metric, the data is smoothed by using LOESS^43^ (LOcal regrESSion), with the span as 1% of the total data points (using MATLAB *smooth* function). By assuming a linear relationship between the motion related parameters and the controller contribution, we computed a linear regression model, which can arguably explain the variance in the contribution of dynamics based controllers (*R*^2^ = 0.52, see Fig. 7a). This suggests that, for the reaching motions with high discomfort values, i.e. more rotations are required and higher musculoskeletal loading is generated, the contribution of dynamics based controllers increases while the contribution of kinematics based controllers decreases. Hence, there is a trade-off between the dynamics and kinematics related controllers depending on the reaching tasks. This trade-off implies that the CNS may utilize a hierarchical control structure. As such, depending on the motion parameters, human motor control first regulates the contribution of each internal model to form a composite model by using a higher level controller. Then, the reaching motion is controlled with respect to this task specific composite model.

To investigate this idea further, we also calculate the discomfort values separately by using individual subject’s data (smoothed as well with span = 5% due to less amount of data). Corresponding values of the coefficients of determination between the discomfort values and *α*_*Dyn*_ for each subject are presented in Fig. 7b. Among 15 subjects, 3 different clusters are observed (Fig. 7b). For the first group, the variation in their motion behavior can be explained by our discomfort metric (*R*^2^ 0.52) better than others. The second group consists of people whose motion behavior can still be accounted for with the proposed model (0.3 < *R*^2^ < 0.5). However, for the third group, the model we proposed fails to capture the variation of motion characteristics (*R*^2^ < 0.3). Two representative subjects are given in Fig. 7c (subject 15) and Fig. 7d (subject 12), where one subject’s data shows similar trade-off compared to the overall results and the other one demonstrates a discrepancy. Possible reasons for the outliers might be, first, the simplicity of our model as the discomfort values we proposed is a linear combination of joint angles. However, the actual metric utilized by the higher level controller might have a different structure, e.g. nonlinear combination of motion parameters. In addition, the distribution *α*_*Dyn*_ possibly depends on other motion parameters besides the joint angles. The second reason could be the fact that subjects show interpersonal variance in their behaviors, e.g. the variance as we observed in the movement duration analysis. Hence, due to the interpersonal variance, it might be difficult to find a single control structure which can explain every subject’s motions.

## Discussion

In this study, we look for the evidence to support multiple internal models hypothesis for a broad range of free space reaching motions. There are two possibilities to consider for the use of different internal models. Either internal models act independently, or they are blended together to achieve a single goal-oriented task. We take the latter claim as our foundation and lay out the analysis of human reaching motion as a bi-level optimization problem. Here, the lower level is treated as a standard OCP, where each OCP with a distinct cost function corresponds to an inverse internal model, and the upper level is utilized as a parameter identification process to compute the contribution of each inverse internal model to the observed motion behavior. In this way, we identify not only the change in the contribution level of different models, but also how this change indicates a trade-off on the utilization of kinematics and dynamics related biomechanical properties, and thus controllers, depending on the initial arm posture and target position. In essence, our analysis suggests a hierarchical control structure, where a higher level controller is responsible for the regulation of the lower level independent internal models.

### Optimal control problem as an inverse internal model

Internal models in sensorimotor integration are empirical constructs to describe learning and control of motor behavior. Even if one assumes that the CNS uses internal models, forward models predicting the arm motion for a specified control has to be distinguished from inverse models that “œcan calculate necessary feedforward motor commands from desired trajectory information”, as Kawato emphasizes^44^. Experimental results supporting both types of models are discussed in literature, cf.^45,46^. As we look into stereotypical motions, one central idea discussed in literature is that, after an initial learning phase, such motions are approximately optimal with respect to an unknown criterion and thus several of optimal control models with different cost functions have been proposed^3–5^. In that regard, optimal control theory provides a mathematical framework to describe learning control processes in biological systems^17,47^. Solving an OCP is to determine *what to do*, i.e. the optimal control signal, in order to achieve certain goals optimally, defined by the cost function, with the given dynamics of the system and a set of constraints. In essence, each OCP with a distinct cost function specifies a unique controller for the movement. This optimal controller -among infinitely many possible controllers- can be considered as an inverse internal model representation within the CNS^21,22,36,44^. Given our formulation and results, human arm reaching motions can be explained as the outcome of a combination of several such optimal controllers and thus multiple inverse internal models. This supports the multiple internal models hypothesis for human arm reaching tasks. Furthermore, such a controller not only finds the optimal control sequence, but also acts as a planner by computing the optimal trajectory. However, note that the control law found by IOC provides an open-loop model, hence there is no feedback consideration. However, as we focus only on stereotypical motions, the effects of sensory update are considered minimal^16^.

### Single vs. multiple internal models

Both MIMs hypothesis and the recently introduced IOC framework rely on the fact that a combination of controllers describe human motor behavior better than single models. One fundamental reasoning for the coexistence of multiple controllers is the CNS’s striking efficiency in learning to control human motions by adapting to changes in the environmental conditions. Each controller is suitable and responsible for one or a small set of motor behavior and depending on the occasion, some of the controllers are appropriately chosen to generate the required motor command. As a result, frameworks with MIMs comprising both forward and inverse models have been introduced, e.g.^2,18,44^. Similarly for (inverse) optimal control framework, since a single model describes a controller for a specific feature of motor behavior, e.g. smoothness or effort, a composite of control models are necessary to capture diverse human movement characteristics simultaneously^33,34,48^. In that sense, our composite OCP formulation can be regarded as a framework for describing and supporting MIMs hypothesis from optimal control point of view. Each OCP is associated with a single cost function, and thus provides a modular structure. Such a modular structure is also in accordance with the motivation behind MIMs. Especially, considering the variety of interaction cases a human may face with (e.g. depending on the object being interacted, the environmental effects, and their possible combinations), such a modular structure allows for providing appropriate motor commands effectively rather than a single controller that needs to take all the external signals into account and solves for control commands on each occasion.

### Forward and inverse internal models

Considering that internal models mimic the transformations between system states, motor commands, and sensory signals, an OCP can be regarded as an inverse internal model^17,36^. In essence, for the stereotypical movements we investigate, solution of an OCP provides the necessary control signals to carry out the reaching task, which is consistent with the inverse internal model idea proposed by Kawato and Wolpert^2^. Even though the bi-level optimization formulation uses the combination of costs in the upper level program to optimize the weighting factors, the lower level program still solves an OCP, for which each constituent OCP model -each associated with a specific cost function- needs to be satisfied simultaneously. Wolpert and Kawato propose a general model to account for motor control’s ability to handle various tasks (e.g. reaching for grasp) concurrently, for which multiple forward and inverse models are paired^2^. As we concentrate on a specific arm reaching task, in our formulation we have a single forward model, i.e. the arm dynamics model that describes how system states evolve given the control input, but it is paired with different inverse models. Hence, the combination of multiple OCPs to control the execution of a single task offers a model-based formulation for the multiple inverse internal models proposed in Wolpert and Kawato^2^, which has been treated in later studies as a black-box function approximator^22,49^.

### Composition of internal models

There have been other studies which focus on multiple models for motor control, learning and adaptation^50^. The focus of these prior work was whether multiple internal models can be learned concurrently^19–21^ and switched depending on the context^51–54^, whereas in our work, free-space stereotypical arm reaching movements are assumed to be already learned, and controlled as a combination of multiple internal models and this combination is sought with a systematic approach by the IOC formulation. A similar study by Davidson and Wolpert analyzed whether two internal models could be learned concurrently for gripping task^21^. They proposed a linear combination of objects’ weights as an internal model representation, and their results verified that dynamic internal models can be additively combined. However, they also acknowledged the necessity for further studies to verify whether similar compositions could be realized for more complex internal models^21^. In our study, a combination of optimal control models (via a composite cost) is proposed which can be applied on tasks which require position and force control at the same time. In addition, it is verified that the composite model explains human movement behavior better than single models, and this compositeness changes with respect to the task parameters.

### Trade-off between dynamics and kinematics

In the controller contribution analysis, we observe a trade-off between the usage of dynamics and kinematics based controllers with regard to the task demands. In theory, an OCP with kinematics related cost functions results in trajectories with high smoothness^3,55^, while the optimization of dynamics related costs minimizes the motor control effort for given reaching tasks^4,56^. It has been acknowledged and verified that motor control involves consideration of different performance measures^17,34,40^. In Liu and Todorov, the focus was on closed-loop control behaviors for reaching tasks, and the change in the control strategy during movement was investigated^57^. However, our main focus is on stereotypical motions for which an open-loop control is assumed, similarly to the prior work in this field^33,34,48^. Composite models were discovered for planar arm reaching motions in a similar work by Berret *et al*.^34^, however, the combination of different models was not analyzed in depth to explain how and why such compositeness is utilized. A similar result was also reported in a recent work by Vu *et al*., where the contribution of the interaction torque to the net torque was found to depend on the load on the forearm, and a trade-off between kinematic and kinetic variables was suggested^40^. Besides, for different arm postures, the musculoskeletal load varies and affects the feeling of comfort^41,42^. This indicates the possibility of the musculoskeletal load as a criterion to describe the trade-off between kinematics and dynamics based models for different reaching tasks. However, an inquiry on how the control models change depending on the task parameters has been neglected. In this work, a discomfort metric is introduced to explain the change in the model contributions with respect to the movement type. In essence, the angular rotations required to execute the reaching task are considered as a representation of the musculoskeletal load. As a result, the trade-off between controllers is revealed by the task demands, and the contribution of dynamics based controllers are found to increase with higher load. For reaching tasks with higher effort requirements, the CNS might be planning the motion depending more on the dynamical effects, while for tasks with low dynamics demands, the smoothness plays a dominant role in the motion planning.

### Hierarchical control

The adaptive usage of smoothness and effort suggests a hierarchical control structure of the CNS, which can be described as a task-estimation phase and an optimal execution phase. For a given reaching task, the CNS first evaluates the motion with respect to a higher level criterion, e.g. the proposed discomfort metric, then based on the estimation, it controls the contributions of different internal models. This phase can be considered as a task-estimation phase which is similar as the responsibility estimator discussed in the MIMs hypothesis^2,44^. However, in prior studies^2,22^, the output control signals from multiple inverse models are linearly combined with a responsibility signal, whereas in our model, even though the weights are linear in the cost functions, which is solved in the upper level program of the bi-level optimization, the underlying optimal control problems and thus their outputs, which are solved by the lower level program, are nonlinear in terms of weights and states, especially in the presence of kinematics, and dynamics constraints. In other words, finding the composite model in an inverse optimal control formulation involves solving the optimization problem that simultaneously minimize multiple costs to satisfy each control model constrained on the dynamical system, i.e. a highly nonlinear, high-dimensional and non-convex solution space. Note that, the estimation relies on personal experience and preference, thus different subjects have different attitudes towards the same task, e.g. the task treated as comfortable for one subject might be uncomfortable for the other subject. This would result in an interpersonal discrepancy during the regulation of internal models. After the task-estimation, the CNS plans and executes the motion with respect to the optimality criterion defined as the regulated contributions of different internal models. This execution phase can be treated as the realization of an OCP. The usage of this hierarchical control structure may help explain how humans achieve optimality in different reaching tasks. Due to the high degrees of freedom of human arm, it is not possible to learn the optimal movement behaviors case by case^2^. It is more likely that, the CNS first learns a set of (optimal) controllers, i.e. inverse internal models, for typical movements, and maintains a functional mapping from task requirements to weighting of these models, similar to the responsibility estimator proposed in Wolpert and Kawato^2^.

### Model fitting

We provide an analysis on the model fitting by using Akaike and Bayesian Information Criterion (AIC/BIC) in the Results section. Here, we also touch upon a couple of points regarding the IOC formulation and the models found. Our analysis shows statistically significant difference, on most cases, between the results of the composite model and the rest considering not only the hand trajectory but also the joint angle trajectory (note that, the inverse problem is solved only on the end-effector (hand) trajectory level, i.e. there is no fitting in joint-angle and/or torque space). In addition, a recent study by Vu *et al*. shows that, even though a single OCP model can match human-like end-effector trajectories, it cannot capture the torque profiles accurately^40^. Hence, it is remarkable that such composite models can predict both kinematics and dynamics related outcomes better than single control models. Additionally, in terms of the fitting quality of the composite model, our bi-level optimization formulation along with a comprehensive greedy search on the parameter space allow for discarding models if necessary, i.e. the weighting factors could be zero. The upper level program (the trust-region based optimizer) in the bi-level formulation provides regularization effect on the weighting parameters, which is commonly applied for inverse optimal control and reinforcement learning problems to prevent overfitting^58,59^.

### Limitations

There are some limitations in the presented work. First, due to the high non-linearity of the IOC formulation, the global optimum is usually not available^35^. To address this issue, we solve the same IOC problem several times and use the best local optimum to approximate the global optimum. However, there is no guarantee about the error between the approximated and the real global optimum. This partially explains the observed variance in the controller contribution analysis. Second, we propose a discomfort metric to simulate the higher level controller utilized in the task-estimation phase. When formulating the discomfort values, the final arm postures are assumed to be known. However, during the task-estimation phase, it is reasonable to think that there is a bias between the estimation and the real posture. This variability might be inherent to movement or internal noise, as there is still on-going debate on whether there exists a Donders’ like law for arm movements^60^. Hence rather than a deterministic expression, the high level controller may utilize a more flexible strategy to minimize this influence. With the proposed simplistic discomfort metric, we intend to present a proof-of-concept formulation for the existence of the hierarchical control structure. It requires further investigation on how the higher level controller is actually formulated, as besides the initial and final arm postures, it might also include other motion parameters or even subjective factors for the given task.

The experiment conducted is designed to allow the subjects to reach their hands to a region rather than a single point target. Hence, our focus is on stereotypical arm reaching characteristics that are observed on the trajectory level, and the corresponding feedforward controllers that might result in such trajectories. We believe this is still an important aspect of motor control, since in our daily lives there are many cases where we interact with our environment in an open-loop fashion, and also it can be seen as an approximation of non-deterministic control due to the stationary environment as well as the fast and stereotypical nature of the motions we investigated. Even though there is evidence for open-loop control of specific tasks in human motor system^61–63^, in general the necessity of feedback control for motor tasks is clear^17^. A combination of both feedback and feedforward processes is likely to be involved for most optimal movement control tasks^45,64,65^. Especially in the context of adaptation to new tasks or new dynamical environments feedback is needed. In that regard, a feedback loop is essential for the analysis of human learning strategies, but the derivation of optimal control strategies is proved to be more complex for the closed-loop approach than for the open-loop one^57^. In essence, we assume that learning processes for such reaching tasks are completed and that “œthe sensorimotor control is best described as being near optimal”^66^ for an unknown cost function subject to the dynamics of the plant. Furthermore, such analysis would also help us better understand and build closed-loop systems to model more complex movement behaviors from control theoretical point-of-view. It is clear that the current IOC formulation can be extended with a non-deterministic formulation and analysis. Considering the signal-dependent noise in motor control, the end-point variance can be tackled with a stochastic optimal feedback controller^5,32,67^. However, integrating stochastic components to an IOC formulation is still an open research question in control theory to effectively solve such problems for complex systems^68,69^.

## Materials and Methods

### Experimental task

During the experiment, participants are required to sit in front of a vertically placed plastic board and perform reaching tasks with their right arm. Nine target areas along with one reference point are marked on the board surface as squares with the side length set to 5 cm. Distance between each target area can be seen in Fig. 2b. The sitting position of the participant is determined by setting the line between the reference point and the center of the shoulder joint vertical to the board surface, and the distance is selected as 80% of the total arm length (see Fig. 1a).

Every participant is asked to reach nine target areas from nine different initial arm postures. While reaching, the participant should hold a fist and use the frontal surface of the fist to touch the target area, in order to reduce the noise caused by finger movements. Before the recording, the initial arm configuration is determined by measuring the joint angles with a protractor. The nine selected initial postures are displayed in Fig. 2. After the measurement, a set of reference tools is utilized to designate the corresponding initial posture. This set of tools consists of two bars with adjustable lengths and positions. The end points of the bars indicate the positions of the elbow and wrist joints for the given posture. The reference tools are placed in appropriate configuration so that during the reaching motion they do not block any potential motion trajectory. The participants are given the following instructions. First, to avoid the decision-making process of target selection, the subjects need to reach the nine target areas in a fixed order as from target one to target nine. Second, the participants should put their arm in the given initial posture as accurate as possible before executing the follow-up reaching tasks. Third, in order to minimize the influence of locating target during the movement, the participants should look at the target area before performing the reaching motion. Fourth, the participants are asked to avoid using the internal/external rotation of the shoulder joint, which is ignored in our arm model. In addition, no instruction about the reaching speed is given, in order to ensure the recorded reaching motions are natural movements.

All participants are trained before the recording to get familiar with the experimental setup and the tasks. If any unintended motion is detected during the experiment, corresponding tasks are executed again. The participants are given enough rest time to avoid fatigue. Each reaching task is performed three times, thus a total of 3645 (9 initial postures × 9 targets × 3 times × 15 subjects) trajectories are recorded. For the IOC calculations, one average trajectory is obtained through the corresponding three trials, hence 1215 IOC calculations are performed in total.

### Data collection

A total of fifteen subjects (11 males, age: 27 ± 4; weight: 67 ± 9 kg, height: 172 ± 5 cm) conducted the experiments and gave their written informed consent for their participation. All participants are right-handed with normal vision ability. None of them received any information about the purpose or hypotheses of the experiment. The study was approved by the ethics committee of the Technical University of Munich School of Medicine. The procedures were carried out in accordance with the relevant guidelines and regulations. The trajectories are recorded by using the multicamera motion capture system Qualysis^70^ with eight cameras at a frequency of 250 Hz. Nine tracking markers are placed on the subject: seven of them are attached to the right arm (three on the shoulder, two on the elbow, two on the wrist) and two to the torso. The position trajectories are smoothed with the built-in filter function of Qualysis. The movement onset and terminal time of each reaching task are selected as the instant at the 5% of the peak velocity of the hand. These 3D position trajectories are utilized in the IOC calculations. The joint angle trajectories are calculated through inverse kinematic technique.

### Model of the musculoskeletal system

A common approach to model the arm dynamics in 3D reaching motions is to consider it as articulated rigid bodies. By ignoring the hand movements, the arm can be treated as a musculoskeletal system which consists of four degree-of-freedoms (DoFs), where the shoulder joint has three rotations and the elbow joint has one rotation. Due to the fact that in our preliminary tests, the internal/external rotation of the shoulder joint is merely activated for the given reaching tasks, it is neglected in the dynamic model. This simplification can increase the computational efficiency of the inverse optimal control calculations while preserving enough accuracy in the results. According to the classical Lagrangian formalism, the dynamics of the 3-DoF arm model can be defined as

1 $${\boldsymbol{\tau }}=M({\boldsymbol{q}})\ddot{{\boldsymbol{q}}}+C({\boldsymbol{q}},\dot{{\boldsymbol{q}}})\dot{{\boldsymbol{q}}}+G({\boldsymbol{q}}),$$

where the variable ***q*** = (*q*_1_, *q*_2_, *q*_3_)^Τ^ denotes the three joint angles and ***τ*** = (*τ*_1_, *τ*_2_, *τ*_3_)^Τ^ represents the torques. Time derivatives of ***q***, i.e. $\dot{{\boldsymbol{q}}}$ and $\ddot{{\boldsymbol{q}}}$, are the joint angle velocities and joint angle accelerations, respectively. *M*, *C* and *G* are the inertia matrix, the Coriolis/centripetal terms and the gravitational vector, respectively. As the viscous frictions and elastic properties of the tissues are difficult to estimate, they are neglected in the dynamics. The upper arm length and the forearm length, as well as the mass, inertia and distance to the center of mass are defined as described in previous researches^48,71^. When the arm is in fully stretched out position, *q*_1_, *q*_2_ and *q*_3_ all have zero configurations. The positive rotation direction of corresponding joint angles are given in Fig. 1b,c. The dynamics is obtained through the AUTOLEV^72^ tool, which is an interactive symbolic dynamics program for formulating motion equations.

### Inverse optimal control problem

The point-to-point reaching motions can be formulated as an optimal control problem^73^ (OCP), which usually has the following form

2 $$\mathop{{\rm{\min }}}\limits_{{\boldsymbol{x}},{\boldsymbol{u}}}\,J({\boldsymbol{x}},{\boldsymbol{u}})\quad {\rm{s}}{\rm{.t}}.\quad \quad {\boldsymbol{h}}({\boldsymbol{x}},{\boldsymbol{u}})=0,\quad \quad {\boldsymbol{g}}({\boldsymbol{x}},{\boldsymbol{u}})\le 0,$$

where *J* is a cost function which represents the optimality criterion of the reaching motion. Usually *J* consists of the vector of system states ***x*** and control signals ***u***. The purpose of OCP is to find the admissible control signal trajectories x^*^ and system state trajectories u^*^ in time *T*, which minimize *J* while satisfying the system dynamics, the boundary constraint ***h***(***x***, ***u***) = 0, and the inequality constraint ***g***(***x***, ***u***) ≤ 0. The system states in this work are defined as ${{\bf{x}}}^{{\rm T}}=({{\boldsymbol{q}}}^{{\rm T}},{\dot{{\boldsymbol{q}}}}^{{\rm T}},{\ddot{{\boldsymbol{q}}}}^{{\rm T}})$. Since the joint torques ***τ*** are smoothly generated by muscle contractions^4^, the control signals are chosen as the time derivative of torques as ${\boldsymbol{u}}=\dot{{\boldsymbol{\tau }}}$. The constraints contain two parts: (*i*) the initial condition ***x***(0) = ***x***_*s*_ and the final condition ***x***(*T*) = ***x***_*e*_ as the boundary constraints, (*ii*) limitations on joint angles ***q***_min_ ≤ ***q***(*t*) ≤ ***q***_max_ as the inequality constraints. The constraints of joint angle velocities and joint angle accelerations are neglected since they are identified to be merely active.

Recent studies show that, the OCP which best describes human behaviors could be a composite of several basic controllers with distinct cost functions^34,40^. From the models proposed in previous literature, we examine four OCP with basic cost functions: the minimum hand jerk^3^ *J*_*HJ*_, the minimum joint angle jerk^55^ *J*_*JJ*_, the minimum torque change^4^ *J*_*TC*_ and the minimum absolute work (energy)^56,74^ *J*_*Enr*_. Then the composite cost function is defined as

3 $$\begin{array}{ll}J={\alpha }_{HJ}{J}_{HJ}+{\alpha }_{JJ}{J}_{JJ}+{\alpha }_{TC}{J}_{TC}+{\alpha }_{Enr}{J}_{Enr}, & \sum _{i}{\alpha }_{i}=\mathrm{1,}\quad i\in \{HJ,JJ,TC,Enr\}\\ {J}_{HJ}={\int }_{0}^{T}{\dddot{x}}^{2}+{\dddot{y}}^{2}+{\dddot{z}}^{2}dt, & {J}_{JJ}={\int }_{0}^{T}{\dddot{q}}_{1}^{2}+{\dddot{q}}_{2}^{2}+{\dddot{q}}_{3}^{2}dt,\\ {J}_{TC}={\int }_{0}^{T}{\dot{\tau }}_{1}^{2}+{\ddot{\tau }}_{2}^{2}+{\dot{\tau }}_{3}^{2}dt, & {J}_{Enr}={\int }_{0}^{T}(\sum _{i=1}^{3}|{\dot{q}}_{i}{\tau }_{i}|)dt,\end{array}$$

where *x*, *y*, *z* are the Cartesian coordinates of the wrist position. ***α*** = (*α*_*HJ*_, *α*_*JJ*_, *α*_*TC*_, *α*_*Enr*_) is a weight vector which represents the contributions of each basic controller. The elements of ***α*** are positive values and sum up to one. For better analysis, we divide the four basic cost functions into two groups, i.e. kinematics related cost functions *J*_*Kin*_ and dynamics related cost functions *J*_*Dyn*_. The corresponding contribution of kinematics *α*_*Kin*_ and dynamics based controllers *α*_*Dyn*_ are defined as

4 $${J}_{Kin}={J}_{HJ}+{J}_{JJ},\quad {J}_{Dyn}={J}_{TC}+{J}_{Enr},\quad {\alpha }_{Kin}={\alpha }_{HJ}+{\alpha }_{JJ},\quad {\alpha }_{Dyn}={\alpha }_{TC}+{\alpha }_{Enr}.$$

Due to different units, each basic cost function has a disparate range of objective values, thus they cannot be equally compared in Eq. (3). To overcome this problem, another scalar factor vector ***S*** = (*S*_*i*_), *i*∈{*HJ*, *JJ*, *TC*, *Enr*} is introduced to balance the objective values to the same range^48^. Hence, Eq. (3) is transformed into

5 $$J=\sum _{i}{S}_{i}{\alpha }_{i}{J}_{i},\quad i\in \{HJ,JJ,TC,Enr\mathrm{\}}.$$

***S*** is obtained through the maximum objective values of individual OCP solutions with respect to each basic cost function. In our experimental data, we find that the minimum joint angle jerk tends to have the largest maximum objective value, therefore the scalar factor of *J*_*JJ*_ is set to 1, then the ratios between the maximum objective values of other basic cost functions and *J*_*JJ*_ are chosen to be the corresponding scalar factors as *S*_*i*_ = *J*_*i*,max_/*J*_*JJ*,max_.

The goal of IOC problem formulation is to identify the composite control model which can best reproduce the observations, specifically the weight vector ***α***. A numerical method for solving an IOC problem is to reformulate it as a bi-level optimization problem^33,34^ presented as

6 $$\begin{array}{ll}{\rm{Upper}}\,{\rm{level}}\,\text{program}: & \mathop{{\rm{\min }}}\limits_{{\boldsymbol{\alpha }}}{\rm{\Phi }}({{\boldsymbol{x}}}_{{\boldsymbol{\alpha }}}^{\ast },{{\boldsymbol{x}}}^{obs}),\\  & \quad \quad \Updownarrow \\ {\rm{Lower}}\,{\rm{level}}\,\text{program}: & \mathop{{\rm{\min }}}\limits_{{\boldsymbol{x}},{\boldsymbol{u}}}J({\boldsymbol{x}},{\boldsymbol{u}}|{\boldsymbol{\alpha }}),\quad {\rm{s}}{\rm{.t}}.\quad g({\boldsymbol{x}},{\boldsymbol{u}})\le 0,\quad \quad h({\boldsymbol{x}},{\boldsymbol{u}})=0.\end{array}$$

The lower level program is a standard OCP which gives the optimal solution ${{\boldsymbol{x}}}_{{\boldsymbol{\alpha }}}^{\ast }$ with respect to a given ***α***. The purpose of the upper level program is to find the optimal weight vector α^*^ which minimizes the distance error between ${{\boldsymbol{x}}}_{{\boldsymbol{\alpha }}}^{\ast }$ and the observation ***x***^*obs*^ measured by metric Φ. However, the recorded Cartesian coordinate trajectories cannot be effectively compared in Φ as they contain uncertainties, e.g. the torso movement and the difference between the subject’s actual arm length and the arm model’s length. To address this problem, we transform the recorded Cartesian position trajectories to the *relative position trajectories in arm model coordinate system* through the following steps:

1. Record the Cartesian position trajectories of the shoulder joint ***t***_*s*_ = (*t*_*s*,*x*_, *t*_*s*,*y*_, *t*_*s*,*z*_), the elbow joint ***t***_*e*_ = (*t*_*e*,*x*_, *t*_*e*,*y*_, *t*_*e*,*z*_) and the wrist joint ***t***_*w*_ = (*t*_*w*,*x*_, *t*_*w*,*y*_, *t*_*w*,*z*_).
2. Derive the observed joint angle trajectory through the arm geometry as ***q***^*obs*^ = *G*(***t***_*s*_, ***t***_*e*_, ***t***_*w*_).
3. Compute the relative end-effector position trajectory in arm model coordinate system by using the forward kinematics of the arm model as ***t***^*obs*^ = *δ*(***q***^*obs*^).

The relative end-effector trajectory ***t***^*obs*^ minimizes the errors caused by different arm lengths and the torso movements, hence it can be utilized in Φ. Based on the feature compared in Φ, two different types of distance metric can be formulated, (*i*) the *joint angle* metric, where the observed joint angle trajectory ***q***^*obs*^ is compared to the optimal joint angle trajectory ${{\boldsymbol{q}}}_{\alpha }^{\ast }$, (*ii*) the *end-effector trajectory* metric, where at first the optimal end-effector trajectory ${{\boldsymbol{t}}}_{{\boldsymbol{\alpha }}}^{\ast }$ is computed by using the same forward kinematics function *δ*, then the distance error is measured between the relative end-effector observation ***t***^*obs*^ and ${{\boldsymbol{t}}}_{{\boldsymbol{\alpha }}}^{\ast }$. In this work we use the end-effector trajectory metric as we find it has a better performance than the joint angle metric. Possible reason is that the three joint angles actually have different degrees of influence on the reaching motions^75^, which may introduce further uncertainty in Φ. Similar problem also occurs when combining the joint angle metric and the end-effector metric, since they have different units and it is difficult to balance them into the same range. The distance error is calculated through the dynamic time warping (DTW) algorithm. In time series analysis, DTW is used for measuring the similarity between two temporal sequences which may vary in speed. The sequences are first warped in the time dimension and then compared to each other.

We utilize the numerical computational toolkit ACADO^76^ to solve the OCP in the lower level program, and a derivative-free optimization method called CONDOR^77^ to find the optimal weight vector in the upper level program. In addition, due to the high non-linearity of the problem formulation, the global minimum is usually not available in the bi-level optimization^35^. In order to get more accurate results, we solve each IOC problem three times with different initial values of ***α***, the one that results in the minimum distance error is considered as the final optimal weight vector α^*^.

### Discomfort metric

The motion parameters of each reaching task can be represented as the initial joint angle configuration ***q***_*s*_ = (*q*_1*s*_, *q*_2*s*_, *q*_3*s*_) and the final joint angle configuration ***q***_*e*_ = (*q*_1*e*_, *q*_2*e*_, *q*_3*e*_). The proposed discomfort metric is a linear combination of six joint angles as

7 $$Discom\,fort={\beta }_{1}\frac{90-{q}_{1s}}{180}+{\beta }_{2}\frac{{q}_{2s}}{180}+{\beta }_{3}\frac{{q}_{3s}}{180}+{\beta }_{4}\frac{{q}_{1e}-{q}_{1s}}{180}+{\beta }_{5}\frac{{q}_{2e}-{q}_{2s}}{180}+{\beta }_{6}\frac{{q}_{3e}-{q}_{3s}}{180},$$

where each parameter is scaled with its approximate joint angle limits and balanced with the normalized weight vector ***β*** = (*β*_*i*_), *i* = 1…6, ||***β***|| = 1. The reason of introducing ***β*** is that, each joint angle possibly has different degrees of influence on the reaching motions^75^. Assuming the discomfort metric can explain the trade-off between the contributions of controllers we observed, the optimal weight vector β^*^ for a given set of reaching motions can be determined by utilizing the contribution of dynamics based controllers *α*_*Dyn*_ through the following processes:

1. For each given ***β***, a set of discomfort values can be derived from each reaching motion as ***Discomfort*** = (*Discomfort*_*i*_), *i* = 1 … *N*, where N is the number of total recorded reaching motions.
2. Along with the set of corresponding values of *α*_*Dyn*_ obtained from IOC calculations as ***α***_***Dyn***_ = (*α*_*Dyn*, *i*_), *i* = 1 … *N*, the coefficient of determination *R*^2^ can be calculated from the data set (***Discomfort***, ***α***_***Dyn***_).
3. The optimal weight vector β^*^ is obtained by maximizing *R*^2^.

Note that, rather than a deterministic mathematical explanation of the relationship between task demand and the contribution of dynamics based controllers *α*_*Dyn*_, the proposed discomfort metric should be treated as a linear regression estimation of this relationship but with multi-dimensional input.

### Model-fitting analysis

For the comparison and analysis of the composite models used to find the best fit to the observed data, we computed Akaike and Bayesian information criterion (AIC and BIC, respectively) metrics. As the IOC formulation provides a deterministic model, residual sum of squares (*RSS*) was used for the computations. Note that, this is only valid under the assumption that the model errors are independent and identically distributed according to a normal distribution. The Shapiro-Wilk test was used to test the normality assumption on the error, and the null-hypothesis was not rejected in any of the cases. The *RSS* values for each model were computed by the dynamic time warping algorithm.

## Electronic supplementary material

Supplementary Material
