# Supplementary material for: An Inverse Optimal Control Approach to Explain Human Arm Reaching Control Based on Multiple Internal Models
Source: Sci Rep. 2018 Apr 3;8:5583. doi: 10.1038/s41598-018-23792-7 (PMC5883007; doi:10.1038/s41598-018-23792-7)
Supplement: Supplementary file 1 — Supplementary Material [file 41598_2018_23792_MOESM1_ESM.pdf]

An Inverse Optimal Control Approach to Explain Human Arm Reaching Control Based on Multiple Internal Models

Ozgur S. Oguz, Zhehua Zhou, Stefan Glasauer, Dirk Wollherr

Supplementary Material

Target	Movement duration (s)	Target distance (m)	Average hand velocity (m/s)	Peak hand velocity (m/s)
T1	1.73 ± 0.36	0.813 ± 0.044	0.53 ± 0.11	1.00 ± 0.23
T2	1.63 ± 0.35	0.775 ± 0.051	0.54 ± 0.12	1.04 ± 0.30
T3	1.70 ± 0.37	0.779 ± 0.058	0.52 ± 0.12	0.98 ± 0.26
T4	1.57 ± 0.32	0.689 ± 0.043	0.48 ± 0.10	0.91 ± 0.22
T5	1.49 ± 0.30	0.645 ± 0.050	0.48 ± 0.11	0.88 ± 0.22
T6	1.57 ± 0.37	0.650 ± 0.060	0.47 ± 0.12	0.86 ± 0.22
T7	1.52 ± 0.30	0.574 ± 0.037	0.41 ± 0.09	0.76 ± 0.18
T8	1.44 ± 0.25	0.535 ± 0.045	0.40 ± 0.08	0.72 ± 0.18
T9	1.47 ± 0.25	0.533 ± 0.052	0.39 ± 0.06	0.72 ± 0.19

Supplementary Table 1. Movement duration and kinematic features of reaching tasks starting from posture 1.

Target	Movement duration (s)	Target distance (m)	Average hand velocity (m/s)	Peak hand velocity (m/s)
T1	1.60 ± 0.34	0.673 ± 0.053	0.46 ± 0.11	0.90 ± 0.24
T2	1.54 ± 0.33	0.625 ± 0.054	0.44 ± 0.12	0.89 ± 0.28
T3	1.65 ± 0.45	0.644 ± 0.060	0.44 ± 0.12	0.87 ± 0.29
T4	1.48 ± 0.35	0.538 ± 0.050	0.40 ± 0.11	0.78 ± 0.22
T5	1.40 ± 0.35	0.485 ± 0.054	0.38 ± 0.11	0.71 ± 0.22
T6	1.48 ± 0.38	0.507 ± 0.068	0.37 ± 0.10	0.73 ± 0.23
T7	1.43 ± 0.31	0.419 ± 0.047	0.32 ± 0.07	0.60 ± 0.15
T9	1.47 ± 0.41	0.383 ± 0.063	0.29 ± 0.09	0.55 ± 0.19

Supplementary Table 2. Movement duration and kinematic features of reaching tasks starting from posture 2.

Target	Movement duration (s)	Target distance (m)	Average hand velocity (m/s)	Peak hand velocity (m/s)
T1	1.54 ± 0.37	0.605 ± 0.043	0.43 ± 0.11	0.85 ± 0.29
T2	1.41 ± 0.35	0.549 ± 0.050	0.43 ± 0.12	0.82 ± 0.29
T3	1.56 ± 0.41	0.555 ± 0.049	0.40 ± 0.11	0.77 ± 0.26
T4	1.44 ± 0.33	0.469 ± 0.042	0.36 ± 0.09	0.70 ± 0.23
T5	1.30 ± 0.31	0.391 ± 0.044	0.33 ± 0.10	0.64 ± 0.22
T6	1.45 ± 0.42	0.408 ± 0.049	0.31 ± 0.09	0.61 ± 0.20
T7	1.38 ± 0.31	0.348 ± 0.040	0.28 ± 0.08	0.54 ± 0.19
T8	1.21 ± 0.26	0.277 ± 0.042	0.24 ± 0.06	0.45 ± 0.14
T9	1.35 ± 0.40	0.276 ± 0.050	0.22 ± 0.06	0.43 ± 0.15

Supplementary Table 3. Movement duration and kinematic features of reaching tasks starting from posture 3.

Target	Movement duration (s)	Target distance (m)	Average hand velocity (m/s)	Peak hand velocity (m/s)
T1	1.68 ± 0.40	0.689 ± 0.061	0.46 ± 0.10	0.87 ± 0.21
T2	1.66 ± 0.42	0.727 ± 0.056	0.50 ± 0.12	0.90 ± 0.21
T3	1.81 ± 0.47	0.845 ± 0.055	0.54 ± 0.13	0.98 ± 0.27
T4	1.52 ± 0.38	0.564 ± 0.062	0.41 ± 0.10	0.75 ± 0.21
T5	1.54 ± 0.54	0.610 ± 0.058	0.46 ± 0.13	0.83 ± 0.24
T6	1.66 ± 0.55	0.746 ± 0.052	0.52 ± 0.14	0.93 ± 0.25
T7	1.47 ± 0.38	0.475 ± 0.060	0.36 ± 0.09	0.64 ± 0.18
T8	1.51 ± 0.43	0.540 ± 0.060	0.40 ± 0.10	0.74 ± 0.21
T9	1.62 ± 0.46	0.669 ± 0.059	0.47 ± 0.12	0.84 ± 0.23

Supplementary Table 4. Movement duration and kinematic features of reaching tasks starting from posture 4.

Target	Movement duration (s)	Target distance (m)	Average hand velocity (m/s)	Peak hand velocity (m/s)
T1	1.54 ± 0.45	0.498 ± 0.077	0.37 ± 0.10	0.68 ± 0.20
T2	1.57 ± 0.51	0.562 ± 0.077	0.41 ± 0.11	0.79 ± 0.22
T3	1.66 ± 0.59	0.709 ± 0.073	0.50 ± 0.13	0.94 ± 0.26
T4	1.43 ± 0.41	0.393 ± 0.066	0.31 ± 0.09	0.59 ± 0.17
T5	1.44 ± 0.49	0.462 ± 0.066	0.37 ± 0.10	0.69 ± 0.20
T6	1.60 ± 0.60	0.630 ± 0.067	0.46 ± 0.12	0.88 ± 0.26
T7	1.41 ± 0.48	0.311 ± 0.063	0.26 ± 0.09	0.48 ± 0.15
T8	1.48 ± 0.52	0.411 ± 0.068	0.32 ± 0.09	0.62 ± 0.16
T9	1.61 ± 0.65	0.570 ± 0.070	0.41 ± 0.12	0.78 ± 0.23

Supplementary Table 5. Movement duration and kinematic features of reaching tasks starting from posture 5.

Target	Movement duration (s)	Target distance (m)	Average hand velocity (m/s)	Peak hand velocity (m/s)
T1	1.51 ± 0.39	0.458 ± 0.064	0.33 ± 0.07	0.64 ± 0.16
T2	1.58 ± 0.44	0.531 ± 0.062	0.38 ± 0.09	0.73 ± 0.21
T3	1.67 ± 0.58	0.673 ± 0.065	0.48 ± 0.12	0.87 ± 0.27
T4	1.44 ± 0.41	0.340 ± 0.061	0.26 ± 0.06	0.50 ± 0.13
T5	1.40 ± 0.44	0.421 ± 0.063	0.34 ± 0.08	0.61 ± 0.17
T6	1.56 ± 0.43	0.601 ± 0.067	0.43 ± 0.09	0.78 ± 0.21
T7	1.35 ± 0.39	0.264 ± 0.058	0.22 ± 0.06	0.40 ± 0.11
T8	1.41 ± 0.50	0.368 ± 0.063	0.29 ± 0.08	0.53 ± 0.17
T9	1.63 ± 0.59	0.544 ± 0.074	0.38 ± 0.10	0.72 ± 0.22

Supplementary Table 6. Movement duration and kinematic features of reaching tasks starting from posture 6.

Target	Movement duration (s)	Target distance (m)	Average hand velocity (m/s)	Peak hand velocity (m/s)
T1	1.66 ± 0.40	0.897 ± 0.056	0.64 ± 0.18	1.22 ± 0.39
T2	1.58 ± 0.41	0.806 ± 0.056	0.60 ± 0.19	1.14 ± 0.42
T3	1.62 ± 0.46	0.719 ± 0.048	0.53 ± 0.17	1.01 ± 0.36
T4	1.61 ± 0.46	0.806 ± 0.056	0.59 ± 0.20	1.16 ± 0.42
T5	1.52 ± 0.40	0.707 ± 0.053	0.54 ± 0.18	1.04 ± 0.41
T6	1.45 ± 0.41	0.611 ± 0.052	0.48 ± 0.16	0.91 ± 0.34
T7	1.56 ± 0.42	0.720 ± 0.054	0.54 ± 0.17	1.05 ± 0.40
T8	1.50 ± 0.38	0.628 ± 0.048	0.48 ± 0.18	0.91 ± 0.35
T9	1.48 ± 0.36	0.524 ± 0.046	0.39 ± 0.12	0.75 ± 0.29

Supplementary Table 7. Movement duration and kinematic features of reaching tasks starting from posture 7.

Target	Movement duration (s)	Target distance (m)	Average hand velocity (m/s)	Peak hand velocity (m/s)
T1	1.65 ± 0.40	0.768 ± 0.055	0.53 ± 0.18	1.07 ± 0.37
T2	1.51 ± 0.37	0.668 ± 0.055	0.50 ± 0.17	1.00 ± 0.37
T3	1.49 ± 0.35	0.587 ± 0.060	0.43 ± 0.11	0.88 ± 0.30
T4	1.52 ± 0.36	0.664 ± 0.057	0.49 ± 0.15	1.00 ± 0.35
T5	1.45 ± 0.34	0.555 ± 0.055	0.42 ± 0.13	0.86 ± 0.32
T6	1.39 ± 0.33	0.467 ± 0.055	0.37 ± 0.13	0.72 ± 0.29
T7	1.48 ± 0.30	0.580 ± 0.056	0.44 ± 0.13	0.86 ± 0.32
T8	1.41 ± 0.32	0.473 ± 0.053	0.37 ± 0.12	0.75 ± 0.28
T9	1.38 ± 0.35	0.365 ± 0.044	0.29 ± 0.11	0.56 ± 0.22

Supplementary Table 8. Movement duration and kinematic features of reaching tasks starting from posture 8.

Target	Movement duration (s)	Target distance (m)	Average hand velocity (m/s)	Peak hand velocity (m/s)
T1	1.66 ± 0.44	0.699 ± 0.053	0.47 ± 0.11	0.92 ± 0.24
T2	1.52 ± 0.38	0.586 ± 0.049	0.42 ± 0.09	0.84 ± 0.19
T3	1.52 ± 0.39	0.501 ± 0.044	0.36 ± 0.08	0.69 ± 0.17
T4	1.47 ± 0.37	0.601 ± 0.059	0.45 ± 0.11	0.90 ± 0.25
T5	1.34 ± 0.32	0.471 ± 0.053	0.38 ± 0.09	0.75 ± 0.19
T6	1.35 ± 0.31	0.365 ± 0.044	0.29 ± 0.06	0.56 ± 0.14
T7	1.44 ± 0.31	0.524 ± 0.065	0.40 ± 0.11	0.78 ± 0.23
T8	1.36 ± 0.40	0.402 ± 0.066	0.33 ± 0.10	0.65 ± 0.19
T9	1.29 ± 0.35	0.263 ± 0.054	0.23 ± 0.07	0.43 ± 0.13

Supplementary Table 9. Movement duration and kinematic features of reaching tasks starting from posture 9.

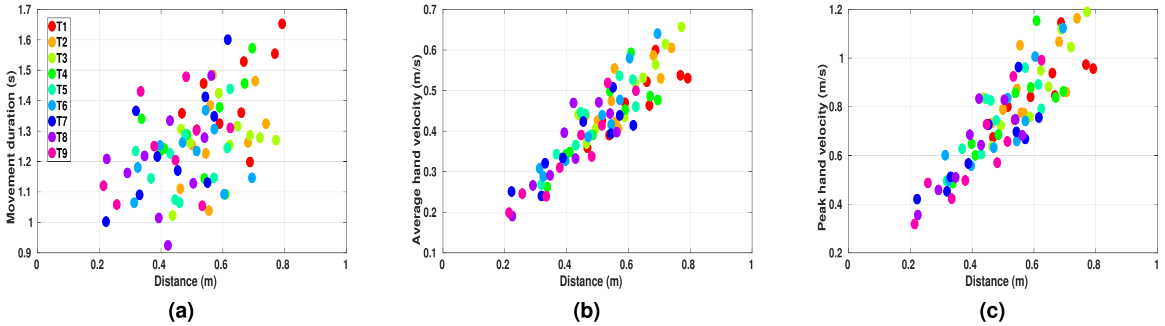

Supplementary Figure 1. Relationship between target distance and movement duration, average hand velocity, peak hand velocity, respectively for subject 1.

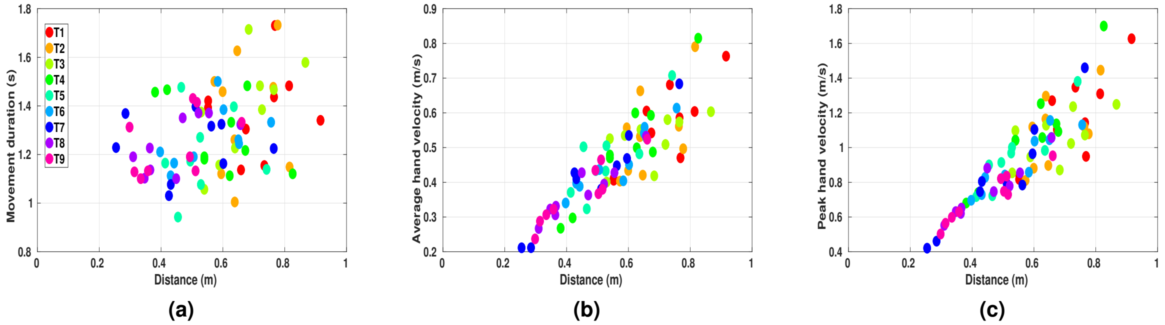

Supplementary Figure 2. Relationship between target distance and movement duration, average hand velocity, peak hand velocity, respectively for subject 2.

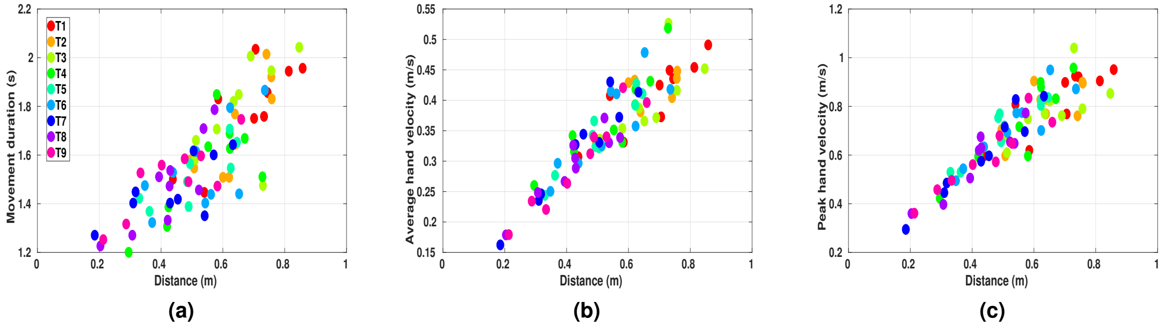

Supplementary Figure 3. Relationship between target distance and movement duration, average hand velocity, peak hand velocity, respectively for subject 3.

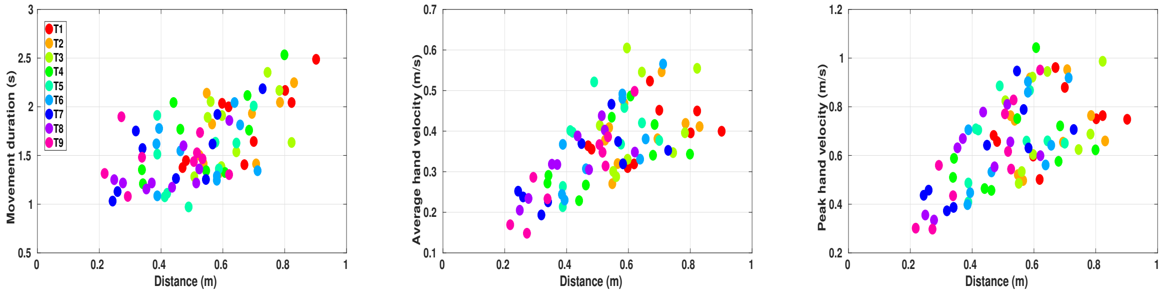

(a) (b) (c)

Supplementary Figure 4. Relationship between target distance and movement duration, average hand velocity, peak hand velocity, respectively for subject 4.

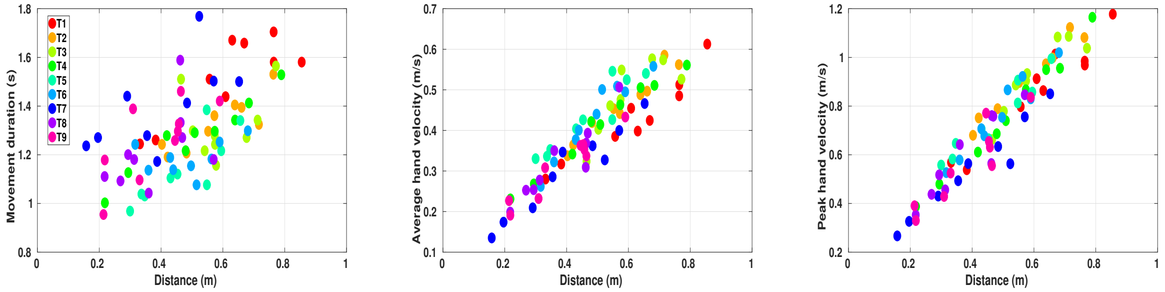

(a) (b) (c)

Supplementary Figure 5. Relationship between target distance and movement duration, average hand velocity, peak hand velocity, respectively for subject 5.

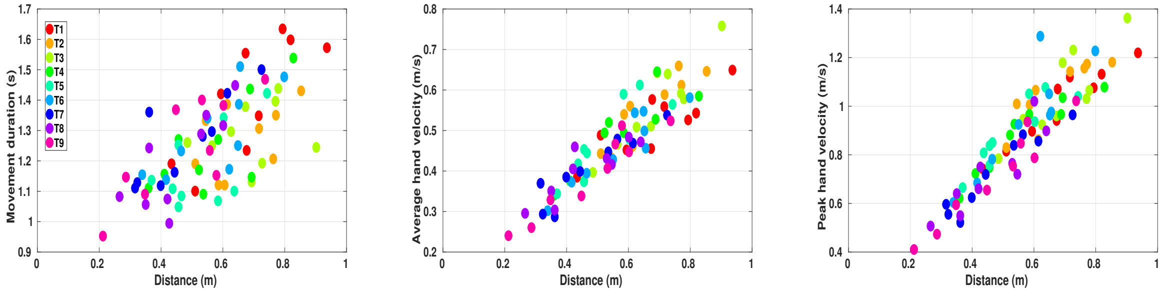

(a) (b) (c)

Supplementary Figure 6. Relationship between target distance and movement duration, average hand velocity, peak hand velocity, respectively for subject 6.

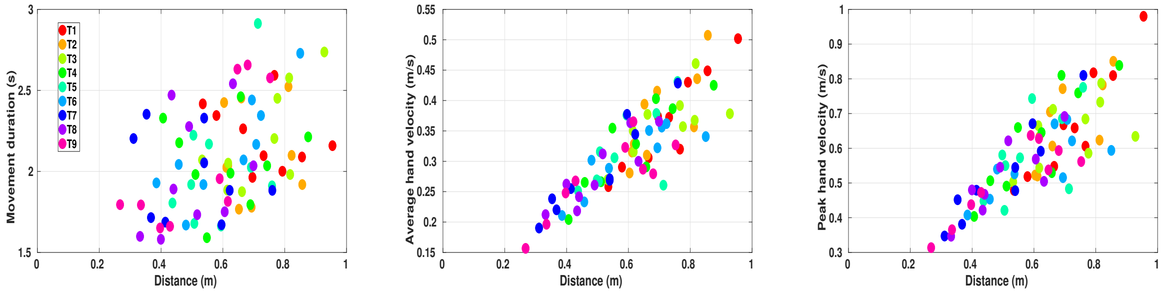

(a) (b) (c)

Supplementary Figure 7. Relationship between target distance and movement duration, average hand velocity, peak hand velocity, respectively for subject 7.

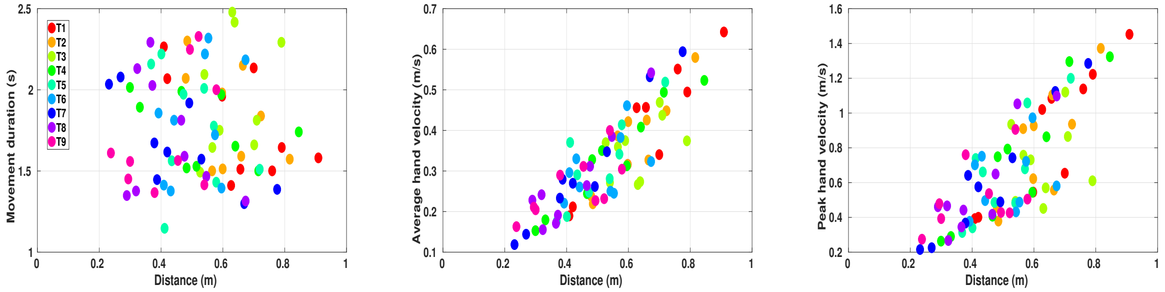

(a) (b) (c)

Supplementary Figure 8. Relationship between target distance and movement duration, average hand velocity, peak hand velocity, respectively for subject 8.

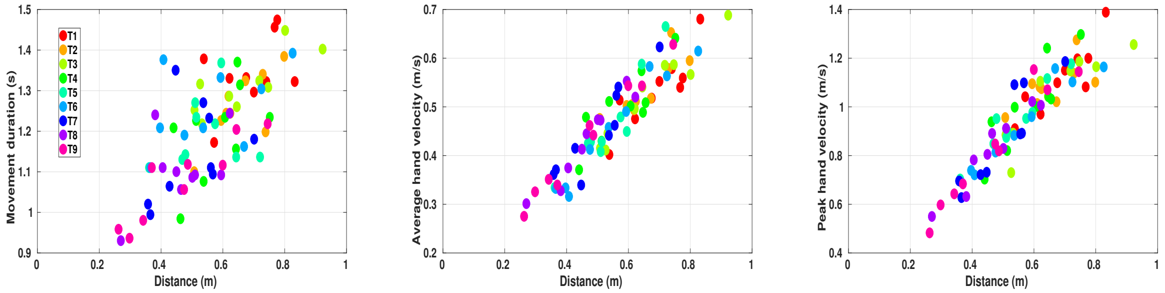

(a) (b) (c)

Supplementary Figure 9. Relationship between target distance and movement duration, average hand velocity, peak hand velocity, respectively for subject 9.

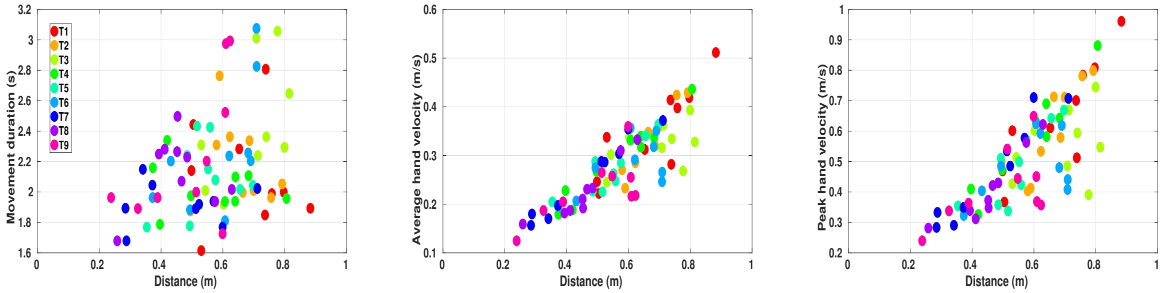

(a) (b) (c)

Supplementary Figure 10. Relationship between target distance and movement duration, average hand velocity, peak hand velocity, respectively for subject 10.

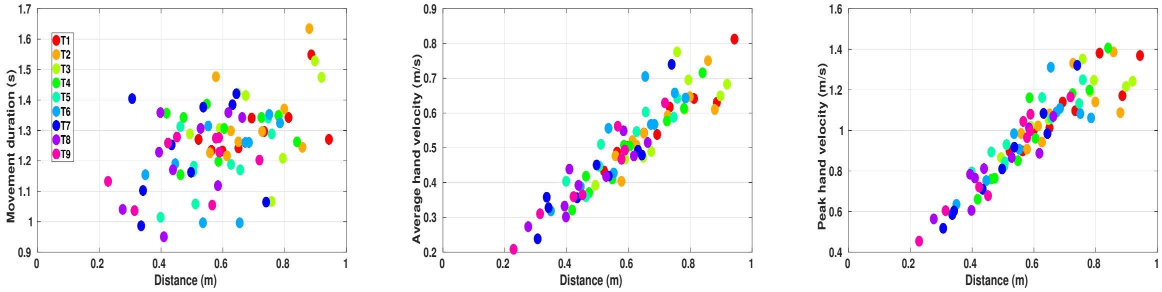

(a) (b) (c)

Supplementary Figure 11. Relationship between target distance and movement duration, average hand velocity, peak hand velocity, respectively for subject 11.

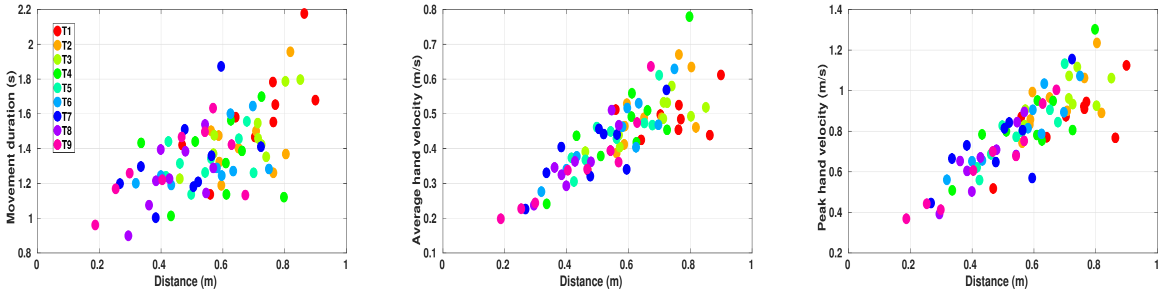

(a) (b) (c)

Supplementary Figure 12. Relationship between target distance and movement duration, average hand velocity, peak hand velocity, respectively for subject 12.

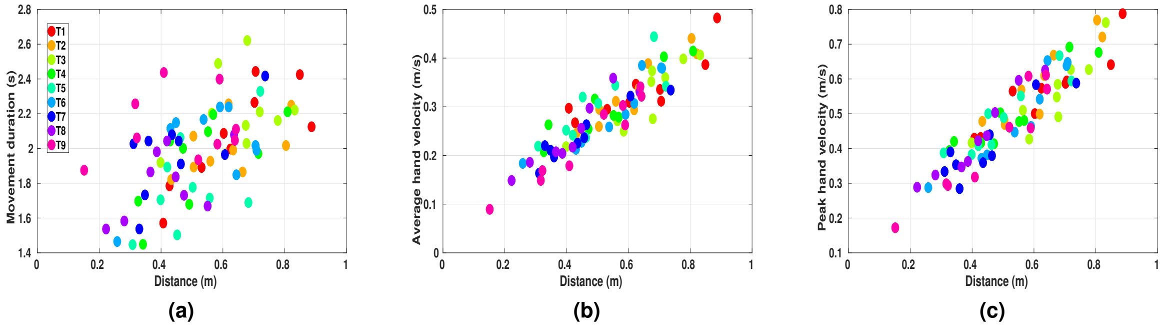

Supplementary Figure 13. Relationship between target distance and movement duration, average hand velocity, peak hand velocity, respectively for subject 13.

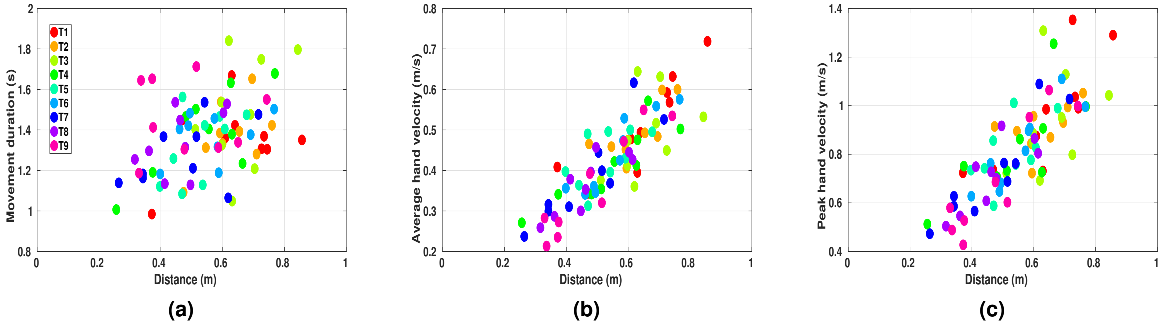

Supplementary Figure 14. Relationship between target distance and movement duration, average hand velocity, peak hand velocity, respectively for subject 14.

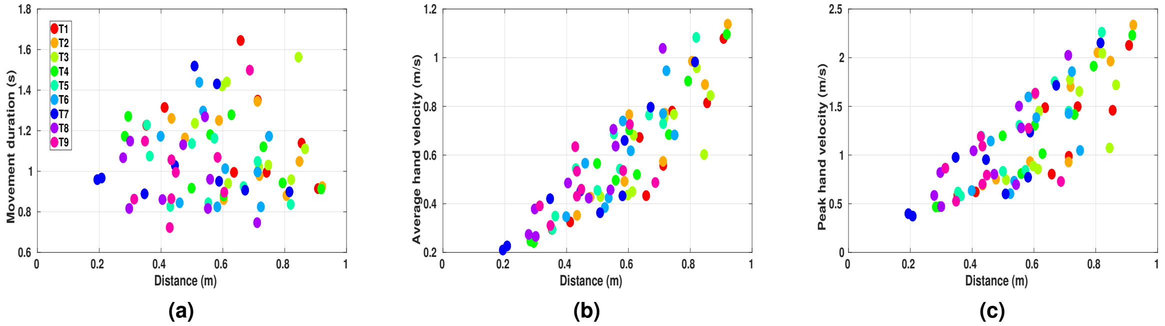

Supplementary Figure 15. Relationship between target distance and movement duration, average hand velocity, peak hand velocity, respectively for subject 15.

Composite model	$Error_{DTW}$ (cm)	AIC	BIC
Joint-angle-jerk + Torque-change	1.43 ± 0.40	8.13	10.30
Joint-angle-jerk + Torque-change + Energy	1.26 ± 0.36	1.76	6.10
Joint-angle-jerk + Hand-jerk + Torque-change + Energy	1.13 ± 0.31	-3.11	3.41

Supplementary Table 10. Model-fitting analysis. Cartesian errors were computed by dynamic time warping (DTW) between each composite model's prediction and the recorded trajectory for the representative subject and normalized over the data points (i.e. 50 discretized timesteps). Values were computed by Akaike and Bayesian information criterion (AIC, BIC, respectively) for the comparison (the smaller values are preferable).

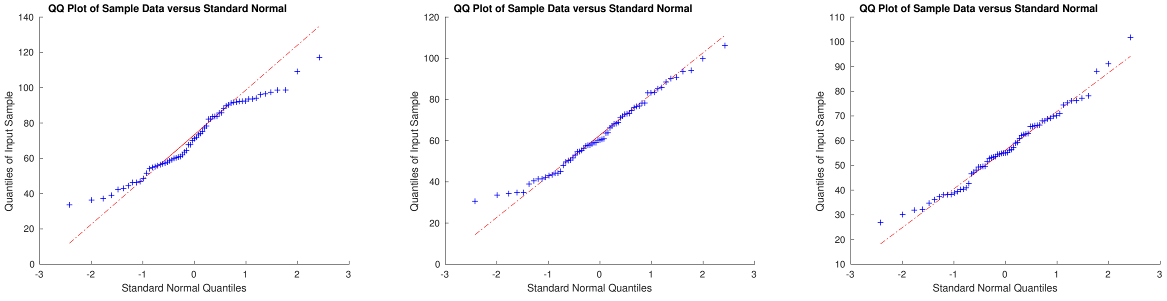

(a) **(b)** **(c)**

Supplementary Figure 16. QQ-plots for the analysis of the residual sum of squares (*RSS*) of the different composite models: (a) 2-cost function, (b) 3-cost function, (c) 4-cost function models. The Shapiro-Wilk test was also used to test the normality assumption on the error, and the null-hypothesis was not rejected in any of those cases.
